# Fragility of foot process morphology in kidney podocytes arises from chaotic spatial propagation of cytoskeletal instability

**DOI:** 10.1371/journal.pcbi.1005433

**Published:** 2017-03-16

**Authors:** Cibele V. Falkenberg, Evren U. Azeloglu, Mark Stothers, Thomas J. Deerinck, Yibang Chen, John C. He, Mark H. Ellisman, James C. Hone, Ravi Iyengar, Leslie M. Loew

**Affiliations:** 1 R. D. Berlin Center for Cell Analysis & Modeling, U. Connecticut School of Medicine, Farmington, CT, United States of America; 2 Department of Pharmacological Sciences, and Division of Nephrology, Icahn School of Medicine at Mount Sinai, New York, NY, United States of America; 3 Department of Mechanical Engineering, Columbia University, New York, NY, United States of America; 4 National Center for Microscopy and Imaging Research, UCSD, San Diego, CA, United States of America; Johns Hopkins University, UNITED STATES

## Abstract

Kidney podocytes’ function depends on fingerlike projections (foot processes) that interdigitate with those from neighboring cells to form the glomerular filtration barrier. The integrity of the barrier depends on spatial control of dynamics of actin cytoskeleton in the foot processes. We determined how imbalances in regulation of actin cytoskeletal dynamics could result in pathological morphology. We obtained 3-D electron microscopy images of podocytes and used quantitative features to build dynamical models to investigate how regulation of actin dynamics within foot processes controls local morphology. We find that imbalances in regulation of actin bundling lead to chaotic spatial patterns that could impair the foot process morphology. Simulation results are consistent with experimental observations for cytoskeletal reconfiguration through dysregulated RhoA or Rac1, and they predict compensatory mechanisms for biochemical stability. We conclude that podocyte morphology, optimized for filtration, is intrinsically fragile, whereby local transient biochemical imbalances may lead to permanent morphological changes associated with pathophysiology.

## Author summary

Podocytes are specialized kidney cells with intricate geometries. Regulation of cell morphology and cell-cell interactions is crucial for podocyte function within the delicate glomerular filtration unit. While the cellular cytoskeleton is dynamic, it must be tightly regulated so that the cell shape and the glomerular filter are maintained. However, genetic defects, disease or drugs may perturb the balance of the cytoskeleton regulators or produce local pressure fluctuations causing proteinuria or focal morphological defects. We derived podocyte morphology from 3-D electron microscopy and constructed a mathematical model to study the actin cytoskeleton. The model reveals the potential for local chaotic cytoskeletal instability when cytoskeleton regulators (Rac1 and RhoA) are imbalanced. We also explore compensatory mechanisms for pathophysiological imbalances in molecular activities.

## Introduction

Podocytes, visceral epithelial cells of the kidney glomerulus, enable the selectivity of the glomerular filtration barrier through their specialized morphology. The cytoskeleton of each highly differentiated podocyte is composed of F-actin, microtubules, and intermediate filaments. All three of these cytoskeletal polymers form the cell body and primary processes, but only actin shapes the foot processes (FPs), the delicate fingerlike projections that interdigitate to form the glomerular filtration barrier [1]. Actin is organized in a spatially specified fashion. Polymerization beneath the plasma membrane gives rise to a cortical actin network, and addition of crosslinkers result in the high density longitudinally aligned bundles, found in the center of the FPs [2]. The FPs establish contact between podocytes and the glomerular basement membrane, in addition to the cell-cell contact via specialized transmembrane junctions called the slit diaphragm, where plasma is filtered [3]. As a result of their highly dynamic filtration function, each FP must withstand tensile stresses (due to glomerular expansion during systole) and transverse shear stresses (imposed by the fluid flow crossing the slit diaphragm), while maintaining contact with the neighboring cells as well as the basement membrane [4, 5]. The loss of the characteristic FP morphology (i.e., foot process effacement) and reduction in the number of podocytes are common hallmarks of chronic kidney disease (CKD), whereby glomerular filtration becomes compromised and protein appears in urine (i.e., proteinuria) [6, 7]. The limited treatment options for CKD are in part due to the sensitive nature of these highly differentiated cells.

Podocytes rapidly dedifferentiate after isolation of glomeruli, losing expression of key slit diaphragm proteins and the specialized morphology within 8 hours, and fully reverting to amorphous epithelial cell morphology within 48 hours [8]. Cultured primary or immortalized podocytes fail to fully differentiate [9]. These observations suggest that the mechanical stresses experienced by the podocyte in the mammalian glomerulus may regulate the integrity of the actin cytoskeleton within the FPs. Without the *in vivo* mechanical stimuli and the final shape signals, cultured podocytes present geometric characteristics (e.g., surface-to-volume ratio, eccentricity, characteristic length, etc.) that are clearly different from the *in vivo* structure [10, 11]. Consequently protein localization, gene expression levels, and active signaling pathways in cultured podocytes are not similar those of the *in vivo* cells [12]. Slit diaphragm has not yet been reconstituted in culture; hence, the unique localization of podocyte specific proteins that lead to the pro-differentiation signaling landscape and the stable actin cytoskeleton of the FPs is absent in cultured podocytes *in vitro* [13, 14]. To understand how the maintenance of the FP morphology and slit diaphragm integrity are regulated, it is important to study the balance of signals within the context of the *in vivo* cell morphology.

Rho GTPases play important roles in the regulation of the actin cytoskeleton [15]. Rac1 promotes the formation of a branched actin network, as found in lamellipodia whereas, RhoA promotes the formation of stress fibers and actin bundles. Expression and activity of both of these GTPases are tightly regulated in the healthy podocyte [16]. For example, while Rac1 knockdown may prevent protamine sulfate-driven FP effacement (a standard animal model of acute podocyte injury), suggesting increased FP stability, Rac1 knockout animals that are subjected to chronic hypertension exhibit FP loss, proteinuria and glomerulosclerosis, showing an opposite effect [17]. Proteinuria and focal FP effacement are also observed when Rac1 is hyperactive [18]. It is not clear how an actin polymerization signaling hub, such as Rac1, may act both as a stabilizing and destabilizing factor under these varying mechanical conditions. It is also puzzling that similar phenotypes could be observed for both Rac1 knockdown and overexpression. A similar outcome is observed with RhoA activity. *In vivo* induction of constitutively active or inactive RhoA damages the actin cytoskeleton causing loss of foot processes and proteinuria [19, 20]. These findings indicate that the levels and activities of both RhoA and Rac1 need to be within a defined range to maintain morphological integrity of the foot processes. What is the quantitative relationship between local morphology and the GTPase regulators of biochemical and biophysical reactions underlying actin cytoskeleton dynamics that stabilize podocyte FPs? To answer this question, we need to develop computationally tractable models based on realistic *in situ* morphologies. We developed dynamical models that combine upstream GTPase signaling with mechanical forces that control the podocyte actin cytoskeleton. Our models account for the actin stoichiometry, exchange between monomeric, filamentous, or bundled states, and the *in vivo* podocyte morphology. To account for the spatial specificity of the complex 3-D geometry of the FPs *in vivo*, we constructed a new quantitative model of a representative podocyte using 3-D serial blockface scanning electron microscope (SBEM) imaging in healthy rats. Our 3-D reconstructed model has sufficient resolution to capture the relationship between volume, surface area and characteristic length of the podocyte geometric features as well as the underlying biochemical and biophysical reactions. Our cytoskeleton model and the spatially specific simulations provide a mechanistic understanding of the *in vivo* observations regarding Rac1 and RhoA dynamics in podocytes, and their relationship to the cytoskeletal stability of FPs. The spatial simulations reveal the emergence of chaotic spatial heterogeneities within the actin cytoskeleton when Rac1/RhoA balance is altered providing a multiscale mechanism for foot process effacement due to propagation of chaotic behavior across FPs. Using this dynamical model, we also show how compensatory mechanisms could impact podocyte cytoskeletal integrity when they appear at different stages of regulatory dynamics.

## Results

### Podocyte morphology

**Fig 1:**
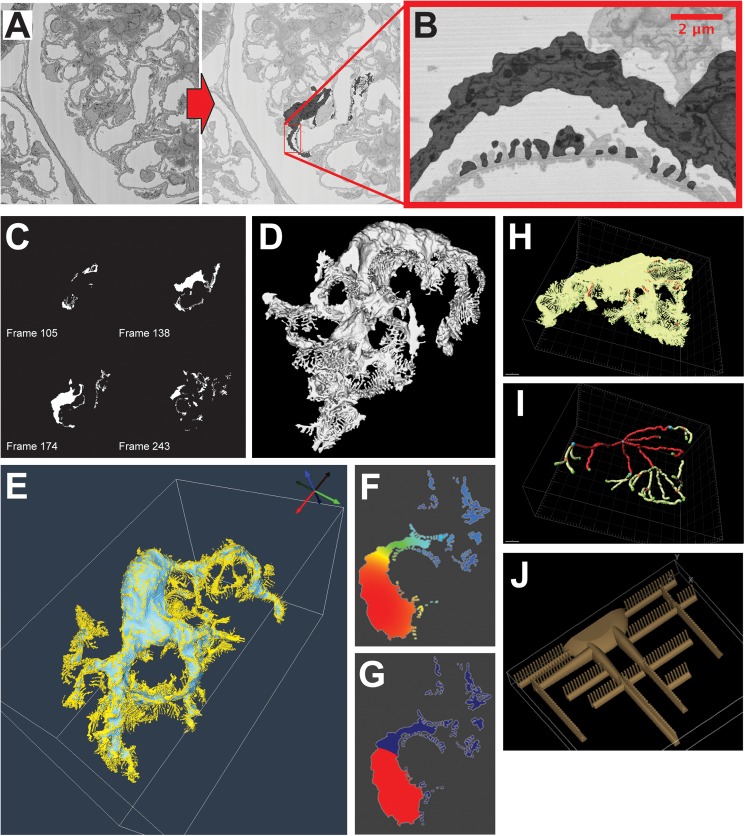
Image segmentation, volume reconstruction, and quantitative analysis of podocyte morphology. **(A)** In order to reconstruct the complete podocyte volume including all the foot processes, we manually segmented stacks of SBEM images. This was done by reviewing the entire stack and identifying all of the projections that emanate from the cell body. **(B)** At full resolution, geometric details of individual foot processes can be seen. **(C)** We then thresholded the segmented images to obtain continuous binary stacks that can be extruded in Rhinoceros and combined in Virtual Cell to form **(D)** a reconstructed 3-D volume. **(E)** We quantified the volume and surface area share of foot processes (FPs) by imposing a Gaussian surface filter in Seg3D, which removed all surface projections smaller than 440 nm. **(F)** We used a commonly used heat transfer model to identify the cell body of the cells: a cytoplasmic specie was uniformly synthesized and allowed to diffuse into the membrane until steady state. Regions with low surface area-to-volume ratios, i.e., cell body, maintain ~90% of the maximum value. **(G)** These sections were assigned as the cell body. **(H)** From the reconstructed volumes, length and angles for major processes and branches were measured using Imaris; the lime colored rendered volume represents the cell volume whereas colored internal lines are the measured paths for the branches. **(I)** For clarity, the internal lines are shown without the rendered volume. Sample branching patterns for two of the cells are shown in supplementary S1 Fig. **(J)** Using the volume, surface area, and branching information, a representative geometry is constructed. For computational simplicity, we assumed symmetry about the xy- and yz-planes, and hereby only half of these are shown. Rat podocytes (RP) used in this figure are RP1, RP8, RP9, RP11, and RP13, respectively, and morphological characteristics of these cells are shown in Table 1.

We reconstructed the *in situ* 3-D geometry of interacting podocytes that would represent core spatial features of a podocyte that permits spatial modeling. SBEM image stacks were processed by manual segmentation and Gaussian filtering to reconstruct podocyte geometry with arborized processes that included nanoscale cell-cell junctions at the foot processes (Fig 1, Supplementary S1 Video). These reconstructions revealed a complex configuration of primary branches emanating from the cell body (Supplementary Material 1). Branching angles and the extent of secondary branching for these processes varied; however, the length and number of processes were similar among different podocytes (S1 Fig); also, despite the large level of deviations in the shape and projection pattern for primary processes, other key geometric characteristics, such as volume, principal dimensions, and surface area of individual podocytes exhibited remarkably low variability between individual podocytes (Table 1). While these studies were in progress, another report used SBEM to reconstruct qualitative features of podocytes [21]. Our reconstruction focused on identifying quantitative spatial parameters that could be used in dynamical models. The results from the analysis of five healthy rat podocytes are summarized in Table 1, with the steps required to achieve this analysis illustrated in Fig 1A–1D.

**Table 1:** Geometric properties, such as surface area (A) and volume (V), of healthy adult rat podocytes are categorized according to different segments of the cell, namely: cell body (CB), major processes (MP), and foot processes (FP) of each rat podocyte (RP). The last column outlines the quantitative morphometric parameters used to generate the idealized, representative podocyte geometry.

Cell	RP1	RP8	RP9	RP11	RP13	Mean	St. D.	Idealized
A_Co+MP_ / V_Co+MP_, μ^-1^	1.8	1.9	1.9	1.5	1.5	**1.7**	**0.2**	**1.7**
A / V, μm^-1^	3.2	3.2	3.4	2.6	3.0	**3.1**	**0.3**	**3.2**
A_FP_/ V_FP_, μm^-1^	8.2	8.6	8.8	8.4	9.3	**8.7**	**0.4**	**9.0**
(A-A_CB+MP_)/A_CB+MP_, %	130	110	130	100	150	**120**	**20**	**140**
V_FP_/V, %	22	19	21	16	20	**20**	**2**	**20**
No of branches	43	30	34	31	34	**34.4**	**5.1**	**36**
Length of branches	10.5	10.4	12.0	11.2	10.7	**11.0**	**0.7**	**-**
V_CB_/V, %	38	33	21	46	50	**38**	**11**	**-**
V_MP_/V, %	40	47	57	38	31	**43**	**10**	**-**
V_Nu_/ V_CB_, %	24	42	42	24	22	**31**	**10**	**-**

We assigned quantitative geometric parameters for podocyte morphology into three distinct compartments based on their reaction-diffusion dynamics: cell body (CB), major processes (MP) that includes primary and secondary processes, and FPs, denoted with appropriate subscripts, respectively. We also reconstructed the nucleus (Nu) although it was not used in the dynamical models. The volume, V, and surface area, A, without subscripts, represent the respective parameters for the whole cell. The CB, MP and FP volumes were derived from the segmented images. Different filters that were based on reaction-diffusion dynamics, allowed reconstruction of a whole cell (yellow surface in Fig 1E) and a “foot process-free cell” (blue surface in Fig 1E). The volumetric properties were computed using Seg3D (Center for Integrative Biomedical Computing at University of Utah, Salt Lake City, Utah, USA) and Virtual Cell (Center for Cell Analysis and Modeling at University of Connecticut, Farmington, CT, USA) (Supplementary Material 1). Boundaries of the different spatial components of the cell were estimated by applying a stratification method that is analogous to a well-known heat transfer problem [22]. The primary processes of a podocyte have a large surface-to-volume ratio in comparison to its cell body. Therefore, a podocyte with uniform volumetric synthesis and diffusion of a given protein within its entire volume and with transport of protein to the extracellular space (proportional to local intracellular concentration) will equilibrate to a much lower concentration at the primary processes relative to the cell body (Fig 1F and 1G, analog heat transfer problem of uniform heat generation with convective boundary condition).

Our analysis revealed that the CB volumes range from 30 to 50% of the total cell volume. FPs correspond to 20% of the total cell volume but constituted nearly 60% of the surface area; the remaining 50% to 30% of the volume corresponds to MP. The distances between the center of the CB and branch points, or ends of the MP, were measured using the filament tool in Imaris (Bitplane, Zurich, Switzerland), as shown in Fig 1H and 1I.

Since the branching parameters such as process length, counts, and distances were widely variable, we utilized the analytical descriptors of segregated volumetric units to construct a representative podocyte. From this analytical geometry, we obtained relevant reaction-diffusion equation parameters (length, surface area, and volume) at the whole cell level. The application of symmetry allowed us to reduce the mesh sizes needed for numerical discretization and the computational cost. FPs emanate from major processes in a symmetrical fashion. Therefore, in our analytical geometry, the major processes are generated as halves, using the sagittal plane as a reflexive boundary condition. The FPs emanate perpendicularly to such a boundary. Since there was no signature branching pattern for these cells, we also imposed axial symmetry. Consequently, the analytically constructed geometry produces simulations of a full podocyte cell, but at a quarter the size (Fig 1J).

The representative geometry was initially built by taking into account only the cell body and major processes. Once the surface-to-volume (see Table 1) and distances of 18 ± 6 μm average distance from centroid to branch point, and 39 ± 2 μm for the three furthest endpoints, satisfied the analysis described above, the FPs were added. The quarter of the cell (CB plus MP) had a volume of 420 μm^3^ and surface area 695 μm^2^. After 233 FPs were added, the final volume was 530 μm^3^ and surface area was 1683 μm^2^ (Fig 1J). Table 1 demonstrates that the volumetric properties of the constructed geometry correspond to a good representation of the analyzed experimentally imaged cells. The surface area of the constructed FPs is on the upper range of the analyzed values. This is likely a conservative estimate; the resolution of the acquired images is of the length scale of the FPs, and a loss of surface detail is expected. The non-spatial computational model described below only uses volumetric variables, and is not affected by this assumption.

### Actin cytoskeleton dynamics in the foot processes

To allow us to understand how actin cytoskeletal dynamics must be regulated to maintain this extraordinary cellular structure, we built a minimal kinetic dynamical model that describes the exchange of actin between monomeric (G-actin), filamentous (F-actin) and bundled states. While the model vastly reduces the complexity of the actual biochemical machinery underlying actin dynamics [23–25], the equations and parameters are approximately related to the key mechanisms controlling polymerization and bundling. For example, a generic “bundling coefficient” is used rather than different expressions corresponding to individual molecular contributors of actin-associated proteins or crosslinkers for bundling. However, different parameters in the model can be related to the activity of Rac1 and RhoA, allowing us to use these as surrogates for the corresponding signaling pathways. Based on experimental observations [26], the actin cytoskeleton in the FP maintains its morphological stability. We used this cytoskeletal model to determine how the balance of GTPase activity affects the spatial stability of FP actin cytoskeleton and consequently its morphology. Initially, we developed an ordinary differential equation (ODE) model. We then mapped the ODE model to the reconstructed podocyte geometry and solved the reaction-diffusion equations for the corresponding partial differential equations (PDEs) numerically.

**Fig 2:**
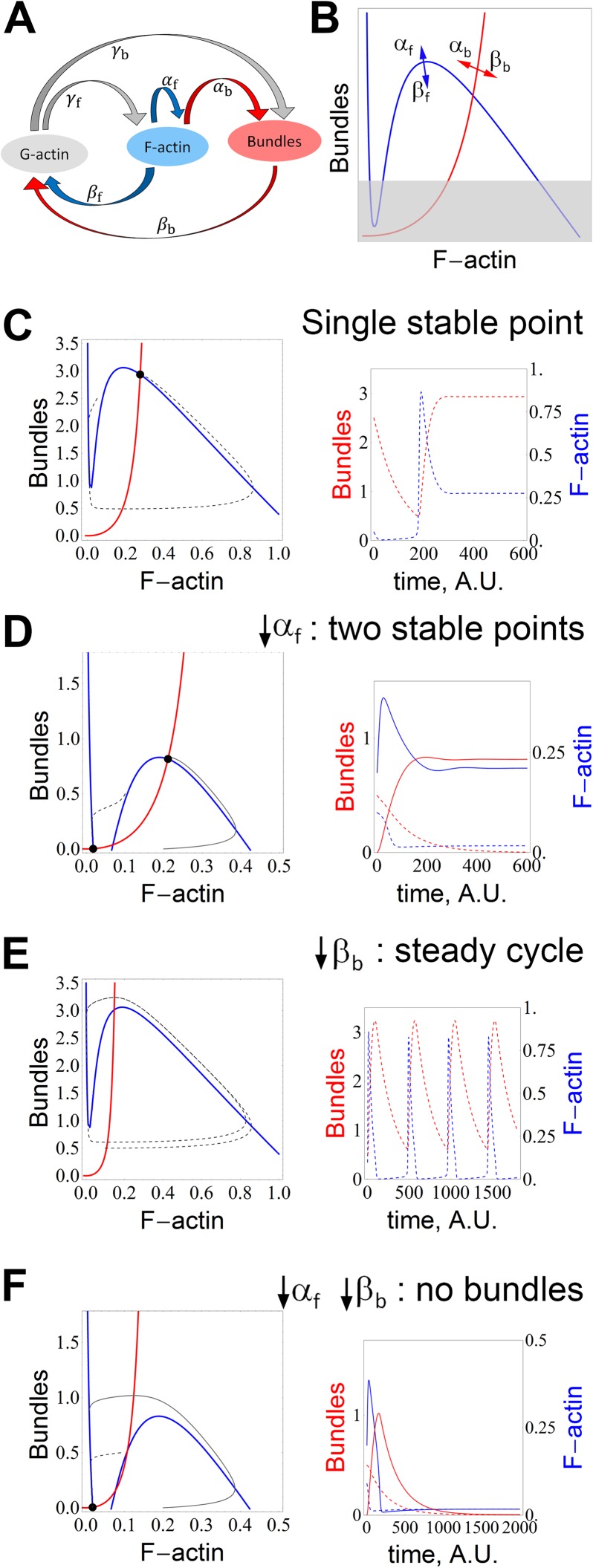
Minimal kinetic model for actin cytoskeleton in FPs. **(A)** Reaction diagram and nomenclature for parameters. Each parameter represents the rate of conversion between the two species marked by the arrows. **(B)** Summary for relationship between nullclines and parameters α_f_, β_f_, α_b_ and β_b_. Blue curves are nullclines for Eq 1 and red curves for Eq 2. The arrows represent the direction of change of a given nullcline when the shown parameter increases. The gray shaded region represents the “effacement region” where actin in FPs does not form adequate amount of bundles and maintain a stable FP morphology. **(C)** A system with strong positive feedback (α_f_, or weak dissociation rate, β_f_) has a single stable equilibrium point. The concentrations for bundles and F-actin in the dashed trajectory of the phase plane (left, dashed gray line) are plotted in the time-series to the right, in red and blue, respectively. **(D)** Weak positive feedback (α_f_) or strong dissociation rate (β_f_) give rise to a second stable equilibrium point, representing the collapse of bundles. The concentrations for the solid trajectory in the phase plane (left) are plotted with the solid lines in the time series (right). **(E)** Weak bundle turnover rate (β_b_) or strong bundling (α_b_) destabilizes the system, and there are no longer stable equilibrium points. However, the cyclic behavior might be able to keep the bundles sufficiently strong at all times. **(F)** A combination of weak bundle turnover rate (β_b_ or strong bundling, α_b_) and weak positive feedback (α_f_) results in complete collapse of the actin cytoskeleton. Model parameters listed in S1 Table. All concentrations are non-dimensional.

Fig 2A shows the relationship between each state and the nomenclature used for parameters. We focused on the three discrete states of the actin cytoskeleton: namely monomeric G-actin, filamentous F-actin, and bundles (i.e. stress fibers), represented by the variables Ga, Fa, and Bu, respectively. The model is described by Eqs 1–3. In Eq 1, the parameter γ_f_ lumps polymer elongation activity (γ_f_*Ga*Fa) and nucleation activity (2*γ_f_*Ga*Ga), as both will produce Fa; for nucleation, the coefficient is necessary for mass conservation since dimerization will form a filament containing 2 actins. The assumption that γ_f_ can represent both elongation and nucleation does not impact the model analysis, as presented in greater detail in the supplementary material (S2 and S3 Figs). The filament state is further enhanced by positive feedback in the first term on the right hand side of Eq 1, which describes nucleation mechanisms that depend on pre-existing filaments, such as Arp2/3-dependent branched nucleation. This term lumps the polymerization triggered by phosphorylation of nephrin and focal adhesion signaling through Nck, N-WASp or Rac1 [27, 28], and represented by a Hill function. Increasing the Hill coefficient does not change the qualitative behavior of the system (S3 Fig). Therefore the parameter α_f_ is non-zero only within the volume representing FPs, where nephrin signaling is localized. A non-linear functional form is necessary to represent both nephrin and small GTPase Rac1 driven polymerization [29–31]. The denominator identifies the high sensitivity region (the positive feedback term is only significant if Fa is at least of the same order of magnitude as the parameter k), and ensures that the effective rate constant is bounded.

In Eq 2, activation of RhoA and crosslinking factors result in the formation of actin bundles. In the first term on the right hand of Eq 2, new bundles are formed by merging filaments while existing bundles grow by addition of filaments, both governed by α_b_. Actin monomers can also be added to filaments within bundles (γ_b_*Ga*Bu). Once a filament becomes bundled, more crosslinks are added and it is unlikely to simultaneously break all connectors between a single filament and the bundle it belongs to. However, there is dissociation of monomers either from depolymerization or biomechanical stress-related rupture, both for filaments and bundles; we represent these, respectively, as -β_f_*Fa and -β_b_*Bu [32, 33] in Eqs 1 and 2, respectively. Eq 3 describes mass conservation within the cell. There are filaments and bundles in all parts of the cell; but we assume that the Ga pool is not strongly affected by dynamics of turnover of the F-actin in the CB and MP, and that crosslinked actin bundles are strongly localized to the FPs [26]. Therefore, we concern ourselves with the FP actin dynamics, where the small volume may lead to large changes in Fa or Bu. Thus, we consider the species Fa and Bu only within the FPs, while Ga freely diffuses to the different regions of the cell, as indicated by the different volume domains for the integrals in the mass conservation equation.

$$\frac{d(Fa)}{dt}=\alpha_{f}Ga\left(\frac{Fa^{2}}{Fa^{2}+k^{2}}\right)+\gamma_{f}Ga\left(Fa+2\;Ga\right)-\alpha_{b}Fa\left(2\;Fa+Bu\right)-\beta_{f}Fa$$ (1)

$$\frac{d(Bu)}{dt}=\alpha_{b}Fa\left(2\;Fa+Bu\right)+\gamma_{b}Ga\;Bu-\beta_{b}Bu$$ (2)

$$\text{Total actin}=\int_{cell}Ga\;dV+\int_{FP}Fa\;dV+\int_{FP}Bu\;dV$$ (3)

Since the exact concentrations of actin at each state in the podocyte are unknown, all of the concentrations in the above equations are set as non-dimensional entities, which would allow quantitative comparison of state spaces. It is known that the ratio of total amount of monomeric actin to polymerized actin in the healthy podocyte is about 1:2 [34]. Here, we use the term “bundle concentration” to represent the concentration of actin molecules in the crosslinked bundle state.

The intersections between the nullclines (lines identifying values of variables that result in zero time derivative) for the variables F-actin and bundles reveal the equilibrium points for the system; increasing any of the parameters presented in Fig 2B will move each nullcline as indicated by the arrows (also in S4 Fig). Bundles are able to sustain mechanical stress better than a filamentous network, and a minimum bundle density is expected to be necessary for the morphological integrity of the individual FP [35]. If the polymerization or bundling rates are not sufficient to overcome the turnover rates, the equilibrium point moves to the “effacement region” of the diagram that is demarcated with gray shading, where loss of FP integrity and specialized morphology (i.e., effacement) is expected.

### The behavior of the non-spatial ODE model reconciles the varied experimental findings on the roles of Rac1 and RhoA

As illustrated by the two trajectories in Fig 2C, under conditions of a parameter regime with a single stable point, the FPs are able to sustain strong bundles irrespective of the initial conditions, or perturbation. This diagram represents ideal circumstances for a healthy stable podocyte. In contrast, reduced filament formation (due to decreased Rac1 activity, or defective Nck signaling) creates a second stable equilibrium point (Fig 2D). If the system moves to this new state, with negligible bundles, the FP morphological integrity will be lost (since this point is located in the effacement region of Fig 2B). Another parametric set that may result in destabilization of the system and loss of FP morphological integrity is via increased bundling (α_b_) or decrease in the turnover of the bundles (β_b_). Depending whether α_f_ is high or low, it may be subject to a cyclic behavior or collapse, respectively (Fig 2E and 2F). The results in Fig 2 correspond to a non-spatial model of actin dynamics, with FPs comprising 20% of the total volume (Eqs 1–3).

The behaviors described by the model are consistent with the experimental observations for response to different activity levels of RhoA *in vivo*. A basal level of RhoA in podocytes (known to activate myosin and promote bundling) has been shown to be necessary for healthy glomerular function [20]. Weak bundling (α_b_) would move the equilibrium point towards higher concentration of F-actin and lower concentration of bundles, shifting the equilibrium point towards the effacement region shown in Fig 2B. In the non-spatial model, the reasons for the damage caused by hyperactive RhoA are not obvious. Our model suggests that RhoA hyperactivity may lead to an imbalance between bundling and depolymerization, where the bundles “consume” all the actin, and the minimum density of filaments required for the positive feedback is no longer achieved. Eventually, the filament density becomes too low and the bundle turnover surpasses its formation. Once this happens, the cytoskeleton may either be subject to temporary (Fig 2E) or terminal collapse (Fig 2F). As the bundles collapse, more monomeric actin becomes available. If the positive feedback for filament growth is sufficiently strong, the filament density recovers and the bundle density increases, resulting in cycles of weaker and stronger bundle density. The instances of weaker bundles may be sufficient to make the FP more susceptible to effacement under increased stress or even under physiological conditions. This scenario is further explored by spatial modeling in the next section.

A weaker positive feedback (α_f_) is the mathematical representation of a system where Rac1 is inhibited. The low α_f_-system in Fig 2D has a stable equilibrium point with moderate bundles. The presence of another stable point, which represents the collapsed cytoskeleton (minimal bundles or filaments), demonstrates that this region of parameter space is not as robust as the system with a single stable point as shown in Fig 2C. In addition, sustained biomechanical stress (e.g., high blood pressure) corresponding to increased β_b_ in the model, moves the bundle nullcline as indicated in Fig 2B, further decreasing the bundle concentration of the stronger stable point. Our model agrees with the physiological observation that, under such conditions, morphological damage may be observed [17].

### Diffusion limited process breaks the oscillatory synchrony, resulting in progressive localized damage

**Fig 3:**
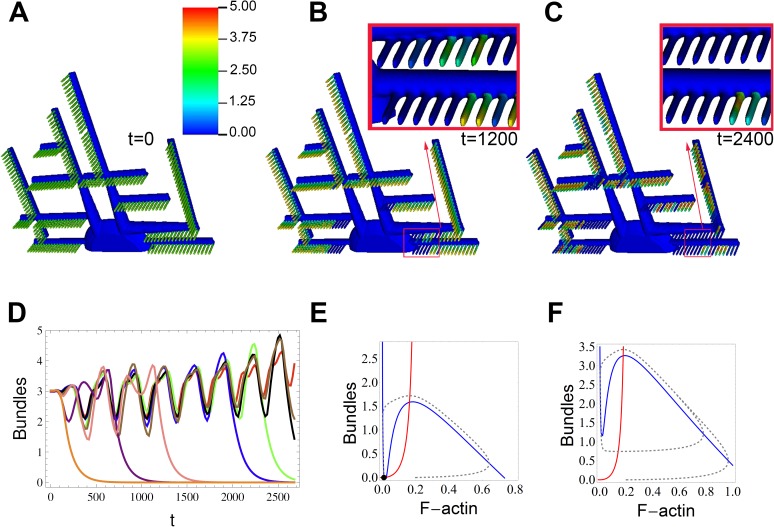
Impact of hyperactive bundling, α_b_, on FP actin stability. Spatially, the cyclic behavior (triggered by sudden, but spatially uniform, increase in α_b_ at t = 40) gives rise to asynchronous and progressive loss of actin bundles within FPs (A-D). **(A)** Spatial steady state actin bundle concentration; simulation parameters are same as those highlighted in Fig 2C. **(B)** Snapshots of bundle concentration in response to increased bundling, at time t = 1200 and **(C)** time t = 2400. Insets correspond to magnified and slightly rotated view of respective boxes. The colorbar represents bundle concentration in normalized arbitrary units. **(D)** Timecourse of bundle concentration at randomly picked FPs, demonstrating asynchronous collapse of bundles. **(E)** ODE solution for increase in α_b_ predicts cytoskeleton collapse when the actin pool is reduced to 70% of that in Fig 2, **(F)** For systems with larger pools of actin (115%), stronger yet still unstable bundles are predicted. In spatial simulations the positive feedback, α_f_, is localized to FPs only, and zero elsewhere.

When we use a partial differential equation (PDE)-based dynamical model with the representative 3-D podocyte geometry shown in Fig 1J, we observed spatial heterogeneities in the oscillatory behavior (Fig 3). This gives rise to permanent localized loss of bundles, leading to effacement of FPs (Fig 3A–3D). As monomeric actin is consumed, it must diffuse from its largest pool in the cell body to all FPs. The varying distance from this major G-actin source to the arrays of FPs, which act as individual G-actin sinks, produce gradients in G-actin, triggering asynchronies in the availability of G-actin at individual FPs. Thus, bundles are collapsing in some FPs, while being strengthened in others. Consequently, as some FPs “release” their pool of G-actin, those monomers are sequestered by neighboring FPs, which are reciprocally reinforced. Fig 3B and 3C show the bundle concentration at different times after α_b_ is increased. After elimination of some FPs, the remaining ones are able to build stronger bundles, temporarily enhancing their ability to overcome stress. However, the system is still unstable and morphological changes would progress slowly. Examination of Fig 3C, in particular, shows that although this is a purely deterministic system, the spatial pattern of bundle loss among the FPs appears to be random. Furthermore, the precise value of the perturbation in α_b_ will produce different patterns. For example, different α_b_ will result in a different steady cycle amplitude range and frequency in the ODE model, which will be translated into a different pattern for the loss of synchrony in the spatial model. Such unpredictable behavior following a change in initial conditions is the hallmark of a chaotic system. Fig 3D shows how the asynchrony can be followed by permanent damage in some FPs. In some of the FPs, bundle concentrations drop permanently to 0, this would correspond to FP effacement. This example illustrates the potential impact of hyperactive RhoA on the FP morphological integrity.

### Localized or transient Rac1 hyperactivity can damage the podocytes

**Fig 4:**
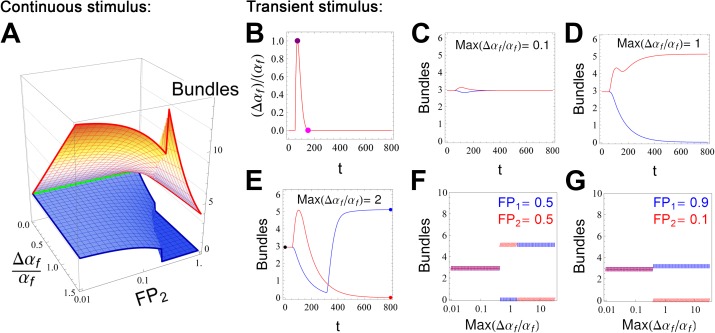
Analysis of bundle stability in the face of spatial differences in positive feedback (α_f_), using an ODE model composed of 2 FP compartments. One FP fractional compartment (FP_2_) is subject to a stimulus that increases actin polymerization by Δα_f_, whereas the remaining fraction (FP_1_) is subject to nominal polymerization conditions, α_f_. This stimulus may either be sustained (shown in A) or transient (shown in B-G). **(A)** A 3-D plot showing steady state bundles on the vertical axis as a function of Δα_f_/α_f_ and FP_2_ (log scale). For this sustained enhancement, the bundle intensity in FP_1_ (blue mesh) and FP_2_ (red mesh) depend on the intensity Δα_f_ and the relative proportions of FP_2_ to FP_1_ (FP_1_ + FP_2_ = 1). As Δα_f_ increases, FPs with stronger feedback form stronger bundles. If the fraction of FPs with enhanced feedback (FP_2_, red mesh) is small, the FPs with normal α_f_ (FP_1_, blue mesh) are unperturbed, while the bundles in FP_2_ are strengthened. Because there is a fixed total amount of actin, stronger bundling in FP_2_ drains actin available for bundles in FP_1_ until a threshold is reached at which collapse of actin bundles in FP_1_ is observed. (**B)** Time course of a transient stimulus applied at t = 40. In C-E, the value of Δα_f_ follows this time course, with varying intensities; equal volume fractions for FP_1_ and FP_2_ were used, with red curves corresponding to FP_2_ and blue to FP_1_. (**C**) A small perturbation allows the system to return to the pre-stimulus steady state. (**D**) When the maximum Δα_f_/α_f_ = 1 (i.e. FP_2_ transiently reaches twice that of FP_1_), a new stable steady state is generated where bundles have collapsed in FP_1_ and increased in FP_2_. (**E**) When the maximum Δα_f_/α_f_ = 2 (i.e. FP_2_ transiently reaches 3 times that of FP_1_) there is a transient increase in FP_2_ bundles followed by collapse and enhanced bundles in FP_1_. The behavior displayed in C, D and E is the hallmark of a tristable system. See the text for an explanation. (**F**). The steady state values for concentration of bundles in fractions FP_1_ (blue) and FP_2_ (red) are shown as a function of stimulus intensity, consistent with C-E. (**G**) Different fractions of FP_1_ and FP_2_ will impact the steady state values (see also S6 Fig).

Each podocyte is subjected to spatially varying mechanical and biochemical signals: it adheres to the basal lamina supporting a segment of the coiled glomerular capillary vessel, and it interacts with several other podocytes. The cell-cell and cell-basement membrane interactions may lead to enhanced Rac1 activity, downstream of transmembrane slit diaphragm protein nephrin or focal adhesions [28, 36]. Using our spatial model, we can demonstrate how this could lead to local damage. The ODE system with parameters as in Fig 2C has a single stable solution. However, this model is only valid if all parameters are uniform among all FPs. To explore and analyze the effect of differential actin bundling activities, we enhanced the ODE model to include two compartments (S2-S6 Eqs). The monomer Ga is assumed to diffuse infinitely fast, resulting in the same concentration in the two compartments. The fraction “FP_1_” of the FPs in compartment 1, retain the original value of α_f_, while there is a localized increase in the positive feedback strength for the complementary fraction of FPs, “FP_2_”, in compartment 2. Fig 4A shows the steady state response for bundle concentrations in such an asymmetric cell, with constant positive feedback values α_f_ and α_f_+Δα_f_ for the fractions of FPs labeled FP_1_ (blue mesh) and FP_2_ (red mesh), respectively. As expected, for small values of Δα_f_, each fraction of FPs have a new concentration of bundles, weaker (FP_1_) or stronger (FP_2_) than when Δα_f_ is 0 (green line). As Δα_f_ and FP_2_ fraction increases, the bundles in the region FP_1_ collapse. Also note that as FP_2_ fraction increases, the total amount of actin becomes a limiting factor, and the local strength for the bundles cannot reach the high values observed in small FP_2_ fractions. This plot also illustrates that even though the machinery for F-actin polymerization is ubiquitous, the regions of the cell that are able to gather stronger polymerizing factors outcompete the remainder of the cell for the primary resource (G-actin), resulting in uneven distribution of F-actin and bundles. This result is consistent with the observation that in podocytes, regulation of the actin cytoskeleton is tightly controlled in the FPs [26], where a high concentration of proteins that mediate actin polymerization are specifically localized [37].

Fig 4C–4E show the impact of a localized transient enhancement (shown in Fig 4B) of the positive feedback on region FP_2_ only (50% of the FPs). If the stimulus is weak, both FP_1_ and FP_2_ regions recover the uniform bundle strength (blue and red lines, respectively, in Fig 4C). For moderate stimulus, FP_1_ fraction loses bundles permanently and FP_2_ reaches a new steady state, with stronger bundles (Fig 4D). For very strong Δα_f_, the initial response is as expected, but at longer times, the bundles in FP_2_ collapse while the bundles in FP_1_ are enhanced Fig 4E). The phase-plane diagrams in S5 Fig help explain the switch. At time zero, either FP_1_ or FP_2_ fraction has the same composition regarding bundles and F-actin. With strong Δα_f_, FP_2_ fraction consumes G-actin in order to develop more and more filaments, while the production rate of filaments for FP_1_ becomes weaker than its turnover. At this point, the two regions of FPs are asynchronous, and their volume fractions and proportions of F-actin and bundles (i.e., their position in the phase-plane) will determine which fraction “wins over” the available G-actin (note the trajectories in Fig 2C). In summary, the steady state response to a localized transient peak in the polymerization positive feedback can either lead to both regions of the FPs to recover the original uniform bundle concentration, or lead to one of the stimulated (FP_2_) or unstimulated (FP_1_) regions to collapse (S6 Fig).

**Fig 5:**
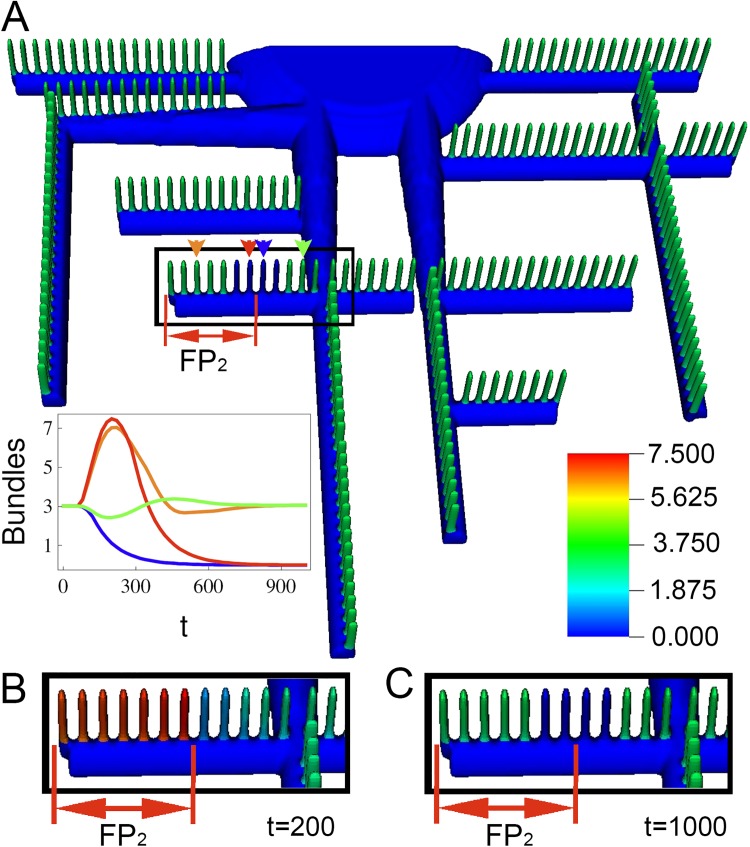
Spatial simulation showing how a transient, localized increase in positive feedback α_f_ in the region labeled FP_2_ impacts actin bundle concentrations in neighboring foot processes. **(A)** Diagram of a region of a podocyte at steady state. A transient jump in α_f_, as in S7 Fig, was locally applied to the region labeled FP_2_ and the state of actin throughout the podocyte was simulated over time. The inset shows bundle concentrations over time in four FPs, two within the FP_2_ segment and two outside it, identified by colored arrowheads and lines. **(B)** Zoomed snapshot of the highlighted region is at time = 200 and **(C)** time = 1000.

The same range of responses was observed in our spatial simulations. Consistent with the results in Fig 4C–4G, the spatial simulations revealed that localized transient increase in the positive feedback α_f_ may lead to localized changes in bundles, both in the regions within and adjacent to the stimulus (Fig 5). Time zero has all FPs in steady state and at same bundle concentration (as in Fig 3A). After time 40, only the region FP_2_ (as marked in Fig 5) is subject to a transient increase in the parameter for positive feedback α_f_, while all other FPs are subject to constant α_f_ (all the other FPs in highlighted region are FP_1_, time series for the feedback parameter α_f_ is shown in S7 Fig). The timeseries plot in Fig 5A indicates the bundle concentrations in FPs identified by the corresponding colored arrowheads. Because the availability of monomeric actin is diffusion limited, a gradient of responses is observed. Within the region FP_2_, there are FPs that recover the original bundle concentration once the stimulus is removed (e.g., the FP indicated by the orange arrow head in Fig 5A). There are also FPs that collapse within the same region (red arrowhead). Interestingly, it is the FPs near the boundary that permanently lose their bundles. Similarly, among the FPs in close proximity to FP_2_ (yet with constant α_f_), some also permanently collapse (blue arrowhead). The 3-D movie of Fig 5 is in Supplementary S2 Video. In the movie, the bounding box identifies the region FP_2_. A second example is shown in Supplementary S3 Video, with a significantly larger region for FP_2_, comprising all FPs within the bounding box.

### Compensatory mechanisms: The earlier, the better

**Fig 6:**
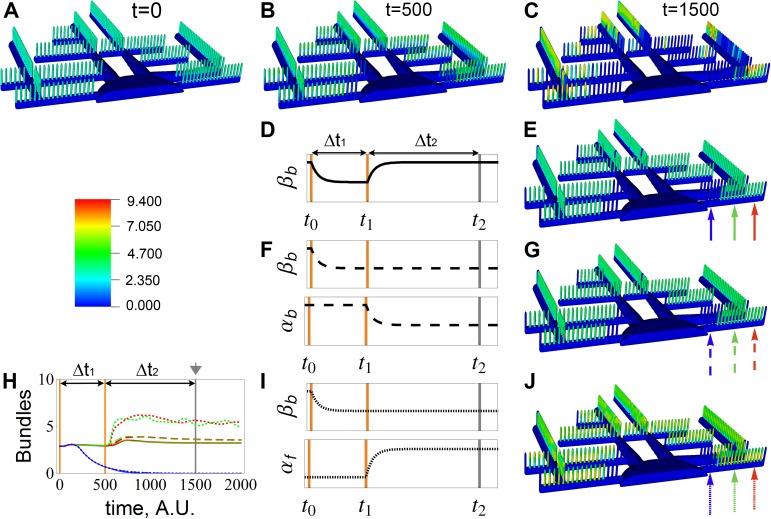
Spatial simulations of the response to perturbed bundling activity, β_b_, highlighting potential compensatory mechanisms for actin instability. **A-C**. Progressive loss of FPs due to a continued decrease of β_b_ imposed at time t = 0; snapshots at time (**A**) t = 0, **(B)** t = 500, and **(C)** t = 1500. **D-J.** Tests of combinations of transient perturbation in β_b_ and α_f_ to explore compensatory mechanisms. **(D)** Return to baseline β_b_ at time t_1_ = 500 results in **(E)** recovery of the majority of the remaining foot process bundle concentrations at t_2_ = 1500. **(F)** Decrease of α_b_ at time t_1_ while holding β_b_ constant results in **(G)** similar stabilization. Finally, **(I)** increase of α_f_ while holding β_b_ constant **(J)** produces similar spatial results. All three interventions prevent progressive effacement (compare C with E, G and J). **(H)** Timecourses for spatial average of bundle concentration in the FPs identified by arrows in snapshots E, G and J (at time 1500, gray arrowhead). Linestyle follows the same pattern as arrows. The same color scale is used for all the 3-D snapshots of bundle concentrations. Parametric perturbations are listed in S1 Table.

Next, we studied the impact of decreasing bundle turnover rate β_b_ and the potential ways to regulate podocyte FP integrity. Bundle turnover rate, β_b_, accounts for depolymerization and damage of bundles due to mechanical stress in the glomerulus. It is expected that under high or low blood pressure, β_b_ would be enhanced or attenuated, respectively. As described above, cytoskeletal stability is dictated by the relationship between the F-actin and bundles nullclines, and as illustrated in Fig 2B, different parameters move the stable equilibrium points in different directions. Initially, the healthy podocyte is in steady state in its single equilibrium point (Fig 6A). By decreasing the bundle turnover rate, the system is destabilized and progressive damage and effacement of FPs are observed (Fig 6B and 6C). Here in this complex geometry, the interplay of Eq 1 and Eq 2, through diffusion of G-actin, produce marked spatial heterogeneity in the pattern of bundles. The trivial compensatory mechanism would be to restore the original value of β_b_ after a certain amount of time Δt_1_ (Fig 6D). Most of the FPs that survived up to the time of the correction (Fig 6B) would have reached stability after elapsed time Δt_2_ (Fig 6E). A similar result is observed if the compensatory mechanism is applied at a later time (S8 Fig). The permanent effacement of several FPs provides a larger pool of actin to be incorporated by the surviving ones, resulting in stronger bundles. This would correspond to the loss of some FPs and the strengthening of others. The timecourse for the bundles in the FPs highlighted by solid arrows is plotted with corresponding solid lines in Fig 6H. The gray arrowhead identifies the time point used for the 3-D snapshots. These spatially asynchronous and irregular timecourses further substantiate the chaotic tendencies of this system.

A second alternative for a compensatory mechanism is the decrease of α_b_, representing decreased bundling coefficient or concentration of crosslinks (Fig 6F). We show that this mechanism can potentially achieve equivalent success as the trivial case of restoring β_b_. Once again, the sooner the intervention, the smaller the population of damaged FPs (Fig 6F and 6G, and timecourse of FPs identified by dashed arrows represented in dashed lines in Fig 6H and S8 Fig).

We also explored the impact of increasing the positive feedback, α_f_, corresponding to hyperactivating Rac1 or other polymerization signals coming from the slit diaphragm (Fig 6I and 6J). Now, high level of polymerized actin is maintained in each of the surviving FPs; however, there is a new mode of oscillation for bundles (and F-actin) along each FP. The simulations suggest that if applied early enough, this compensatory mechanism may help keep the FP morphology intact (Fig 6J). Similarly to the previous cases, the surviving FPs are able to build stronger bundles, and in spite of the oscillatory behavior, equivalent number of FPs seem to be preserved in comparison to the previous examples (Fig 6J, 6E and 6G). The overall conclusion is that there are several potential compensatory mechanisms that may be activated to restore FP stability, and the sooner the regulatory response is activated, the larger the number of surviving FPs.

## Discussion

We acquired 3-D electron microscope imaging data of rat kidney glomeruli, individually segmented podocytes, and quantitatively analyzed their morphological features to determine the average volume and surface area of the cell body, major processes, and foot processes. This resulted in construction of a representative 3-D podocyte model that is amenable to PDE-based reaction-diffusion modeling with physiologically relevant mechanisms. To the best of our knowledge, this is the first time such a detailed quantitative reconstruction was ever achieved. The variability in the geometrical parameters measured between the five cells of Table 1 was remarkably small. Through our morphometric analyses, we show that the FPs corresponded to 20% of the volume of the podocyte, while doubling its surface area. Consequently there is a large pool of diffusive monomeric G-actin that is available for the FPs. However, the G-actin pool is not instantly or uniformly available: the monomers must diffuse across the different regions of the cell. Our representative podocyte geometry carefully preserved the length-scales, volumetric and diffusive properties, quantity and distribution of FPs, primary processes, and cell body, thus enabling physiologically relevant simulations. The range of spatiotemporal dynamics within the real geometries of podocytes is likely to be broader than this idealized geometry, but we feel that this idealized geometry will be representative; it also has the advantage of being more computationally tractable, both in simulation time and for analysis of simulation results.

To explore the consequences of the unique podocyte morphology on the maintenance of cytoskeletal integrity of the FPs, we developed a minimal model of actin dynamics and compared non-spatial (ODE-based) simulation results with full reaction-diffusion (PDE-based) models in the constructed geometry. The minimal model represents key regulatory mechanisms controlling actin polymerization and filament bundling. It contains positive feedback in actin polymerizations, representing Arp2/3-dependent branching nucleation on preformed mother filaments. This positive feedback results in regions of parameter space that produce oscillatory F-actin and bundle kinetics in the ODE simulations (Fig 2). Another mechanism that would be similarly represented by this positive feedback is actin polymerization downstream of nephrin phosphorylation and Nck localization to the FP slit diaphragm. However, it should be emphasized that we did not model the detailed biochemistry regulating actin polymerization and bundling, some of which is still unclear. Rather, our model uses a reasonable level of biochemical specification that allows us to focus on the influence of the complex geometry of the kidney podocyte.

Spatial simulations explicitly consider the process of diffusion of G-actin in and out of FPs from the large reservoir of the cell body and primary processes. The interplay of this diffusion and the positive feedback inherent in the actin dynamics can produce sharp regional differences in the level of actin bundling within closely neighboring FPs. Without bundled actin, the FP would be resorbed into the parent process leading to effacement. Therefore, we assumed that the loss of bundles in FPs leads to loss of structural integrity. Even when a change in bundling activity is distributed uniformly throughout the cell, an apparently chaotic, spatiotemporal pattern of bundle formation and collapse is observed in the FPs (Fig 3). One of the major unanswered questions in podocyte biology is the source of the inherent heterogeneity in podocytopathies. Diseases, such as focal segmental glomerular sclerosis, affect only a subpopulation of podocytes with a large spatial variability in disease etiology. Based on our spatial dynamical model simulations, we suggest that even global changes in stress (e.g., due to hypertension) can lead to selective effacement of FPs with large spatial variability, which may explain the pathophysiological disease progression of numerous podocytopathies.

The balance of Rac1 and RhoA activity (that is represented respectively by actin polymerization and bundling in the model) needs to be tightly regulated to maintain FP stability. This is all the more critical in FPs, where these molecules may be present at low copy number and result in potential stochastic instabilities. Stress (whether focal or global, transient or continuous) can lead to spatially heterogeneous chaotic behavior, and ultimately, to irregular heterogeneous patterns of bundle collapse in FPs. Fig 4 shows how transient activation of Rac1 (i.e., actin polymerization) in one collective region of FPs can shift the steady state balance between F-actin and bundles in all regions. A much more localized transient perturbation, as shown in the spatial simulation of Fig 5, can produce *permanent* changes in the bundle distribution within the system, both within the perturbed region and its immediate vicinity. Unfortunately, experimentally accessible live podocyte models that retain this cell’s unique morphology are not yet available. When this experimental problem is solved, direct manipulation of the regulatory mechanisms (e.g. using local photoactivation of RhoA or Rac1 [38]) will help to test whether the chaotic responses predicted by our model can be validated.

Direct damage to FPs, due to high blood pressure for example, can be captured in the bundling turnover parameter β_b_. Changing β_b_ globally, but transiently, can produce permanent changes in bundle distribution (Fig 6A–6C); the severity of FP effacement (represented by loss of bundles) is directly related to the duration of the perturbation. It is possible that regulatory mechanisms could be modulated to alter Rac1 and RhoA activities in response to stress to ameliorate the imbalance. The behavior of the system under three potential compensatory interventions in response to lowered β_b_ are explored in Fig 6D–6J. These, too, could be tested experimentally once an appropriate *in vivo* or culture preparation is established.

The model for the actin cytoskeleton of the podocyte FP provides a framework for understanding recent findings on the *in vivo* activity levels of the small GTPases RhoA and Rac1. Our model captures the need for a balance between polymerizing, bundling, and turnover rates for the actin cytoskeleton. Our results are consistent with the observations that a minimum level of RhoA is necessary; however, hyperactivity results in destabilization and progressive loss of FPs [16]. We propose that upon inhibition of Rac1, the cell may sustain its FP integrity with sufficient levels of actin bundles. Signaling through nephrin stimulates actin polymerization, which could also influence the parameter α_f_; similarly, mutations in crosslinking proteins would impact the parameter α_b_. Changes in either of these positive feedback parameters for polymerization may give rise to a second equilibrium point for the system, decreasing its robustness.

We hypothesize that healthy cells present a set of parameters that result in a single equilibrium point, representing FPs with strong bundles. In such circumstances, all FPs are stable and have identical properties. Using a reconstructed geometry, we studied the impact of parametric inhomogeneity. Simulations using a representative geometry with features that are important for the diffusion process (such as distances, cross sectional areas and surface area-to-volume ratios for FPs, primary processes and cell body) were used to study the crosstalk between G-actin whole-cell diffusion and the localized polymerization of F-actin in FPs, followed by crosslinking into bundles. As demonstrated here, G-actin availability was a limiting factor, and strong localized polymerization may disrupt the cytoskeleton of FPs elsewhere. The diffusion limited G-actin availability may also disrupt otherwise synchronized oscillations. This results in slow and progressive loss of bundles, the surrogate for effacement of FPs. Of course, the sudden localized collapse of actin structures may be driven by a local inhibitory effect (either decreased polymerization rate constant or enhanced turnover). However, in this work, we demonstrate the feasibility of an alternative hypothesis: enhancing polymerization in remote regions of the podocyte, by a number of signaling pathways, may sufficiently disrupt the balance of G-actin availability to irreversibly drive the local collapse of existing FPs.

## Materials and methods

Methods for processing of podocyte images and analysis of podocyte morphology are detailed in *Supporting Material*. The analysis of the ODE model was performed in Mathematica (Wolfram) and the spatial simulations of PDEs in Virtual Cell (VCell.org). The full VCell model is named “Falkenberg:PodocyteStability” and may be accessed through vcell.org. Segmented and reconstructed SBEM imaging data can be obtained through Dryad data repository at doi: 10.5061/dryad.09d0k. Further details of computational methods and analyses are presented in Supporting Material.

## Supporting information

S1 Fig **(A)** Branching patterns with nodes (labeled in boxes) and distances (numbers over arrows) for two different cells. Node ‘1’ corresponds to the center of the cell (core). **(B)** Histograms for the distances of individual branches of primary and secondary processes for the five adult rat podocytes showed remarkable similarity.
(TIF)

S1 Table Parameters used in model for each figure, as in Eqs 1–3. The parameter k = 0.22. Values differing from the ones used in Fig 2C are in bold. All units are arbitrary.
(PDF)

S2 Table Nomenclature used in the spatial VCell model. (PDF)

S2 Fig Comparison of phase plane diagrams for different values of parameters where ‘n’ represents actin nucleation coefficient and the Hill coefficient ‘h’ represents the positive feedback for F-actin polymerization. While the locations of the equilibrium points are altered, the qualitative relationship between the nullclines is unchanged.
(TIF)

S3 Fig As in the model of the main text, hyperactive bundling, α_b_ (value 0.05 in this figure vs. 0.03 in S2 Fig) will either destabilize the bundles or cause their total collapse. (TIF)

S4 Fig Consistent with Fig 2C and 2D, decreasing the parameter α_f_ from 0.32 (Fig 2C) to 0.1 (Fig 2D) will shift the system from having a single stable point (2C) to having three equilibrium points (two stable and one unstable, 2D). Other parameters as indicated in S1 Table.
(TIF)

S5 Fig **(A)** Time course for transient stimulus imposed on the positive feedback α_f_ for fraction FP_2_ or all FPs, and trajectories for concentrations of F-actin and bundles in the foot processes corresponding to regions FP_1_ (constant α_f_) and FP_2_ (transiently stimulated). **(B)** Trajectory for FP_1_. The time point of the peak and end of stimulus are represented in purple and magenta, respectively, in all plots. Time point zero is in black (at identical concentrations for FP_1_ and FP_2_) and steady-state value for each fraction is represented by shades of blue. **(C)** Steady state bundles in fractions FP_1_ (blue) and FP_2_ (red) as a function of stimulus intensity. **(D)** Trajectory for FP_2_. The time point of the peak and end of stimulus are represented in purple and magenta, respectively, in all plots. Time point zero is in black (at identical concentrations for FP_1_ and FP_2_) and steady-state value for each fraction is represented by shades of red. The intensity of the stimulus will alter the relative position between the two trajectories for unstimulated (FP_1_) and stimulated (FP_2_) fractions. Consequently, for sufficiently large perturbations, either region may collapse.
(TIF)

S6 Fig Steady state concentrations of bundles in unstimulated (FP_1_, blue) and transiently stimulated (FP_2_, red) fractions of FPs as a function of stimulus intensity. Over a broad range of fractions of FP_1_ and FP_2_ either region of the cell is subject to damage (collapse of bundles) if the perturbation is sufficiently strong.
(TIF)

S7 Fig Virtual Cell plot showing time course of the parameter α_f_ in region FP_2_ (purple) and region FP_1_ (light brown). The spatial results for bundle concentration are shown in Fig 5. Nomenclature for parameters is described in S2 Table.
(TIF)

S8 Fig Investigating possible compensatory stimuli against progressive loss of actin bundles within FPs. **(A)** Initial concentration of bundles at t = t_0_ where β_b_ is reduced. The result is heterogeneous loss of bundles in some FPs at times **(B)** t = t_0_ + 500 and **(C)** t = t_0_ + 1500. Three lower rows of panels show the three different scenarios under which the bundling could be modified after a finite time, t_1_ following injury: **(D)** the parameter β_b_ recovers its original value and the stabilized FPs can be observed after **(E)** t_1_ = 500 or **(F)** t_1_ = 1500. **(G)** Parameter β_b_ can be decreased to compensate after t_1_ and stabilized FPs can be observed at **(H)** t_1_ = 500 or **(I)** t_1_ = 1500. **(J)** Alternatively, increase in α_f_ can also halt loss of bundles in FPs whereby stabilized FPs can be observed at **(K)** t_1_ = 500 or **(L)** t_1_ = 1500. We can visualize the timecourses for bundle concentrations in randomly selected FPs (as identified by color-coded arrows) at **(M)** t_1_ = 500 or **(N)** t_1_ = 1500. Line style follows the same pattern as arrows, and corresponds to value of a single voxel in the middle of the corresponding FP. All 3-D snapshots follow the same color scale shown in bottom left (except for L, represented with skewed scale in parentheses). Under all of these scenarios, an earlier intervention leads to markedly improved homogeneous restoration of bundles. This can be clearly seen by the difference between the early intervention within the middle column (E, H, K) and late intervention within the right column (F, I, L).
(TIF)

S1 Video 3-D rendered rotating view of three neighboring rat podocytes. (MOV)

S2 Video Time course of FP bundle concentrations after local transient modification of bundling as shown in Fig 5. (MPG)

S3 Video Time course of FP bundle concentrations after local transient modification of bundling on a larger region. (MPG)

S1 Dataset Nodes and relative branch distances for the five rat kidney podocytes. (PDF)

S1 Supplementary Materials and Methods (PDF)

S1 Text Description of data deposited to Dryad. (DOCX)
