# Supplementary figures and images for: Fragility of foot process morphology in kidney podocytes arises from chaotic spatial propagation of cytoskeletal instability
Source: PLoS Comput Biol. 2017 Mar 16;13(3):e1005433. doi: 10.1371/journal.pcbi.1005433 (PMC5373631; doi:10.1371/journal.pcbi.1005433)

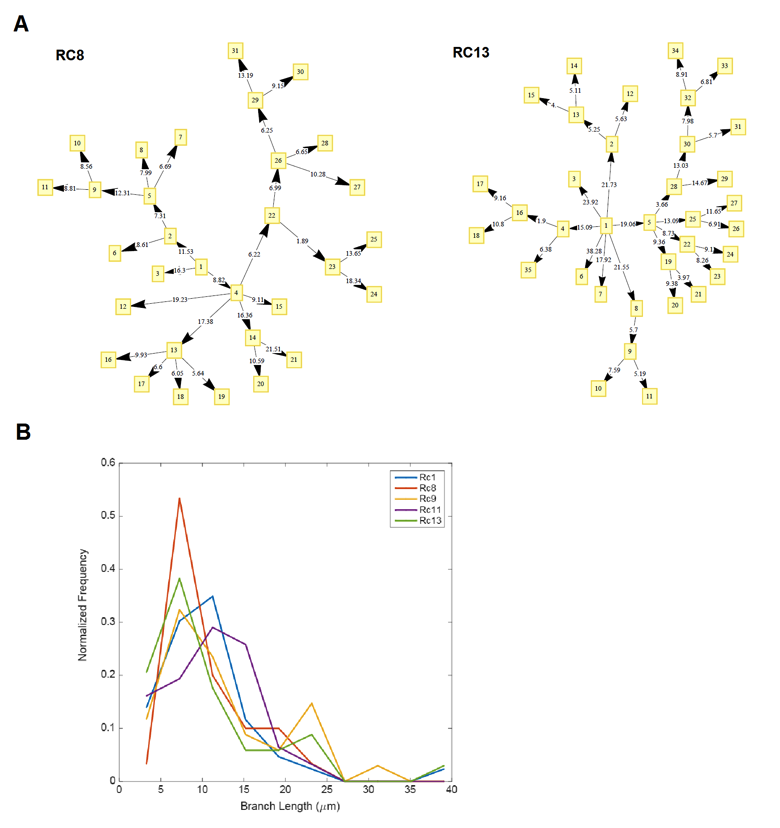

Supplement: S1 Fig — (A) Branching patterns with nodes (labeled in boxes) and distances (numbers over arrows) for two different cells. Node ‘1’ corresponds to the center of the cell (core). (B) Histograms for the distances of individual branches of primary and secondary processes for the five adult rat podocytes showed remarkable similarity. (TIF) [file pcbi.1005433.s001.tif]

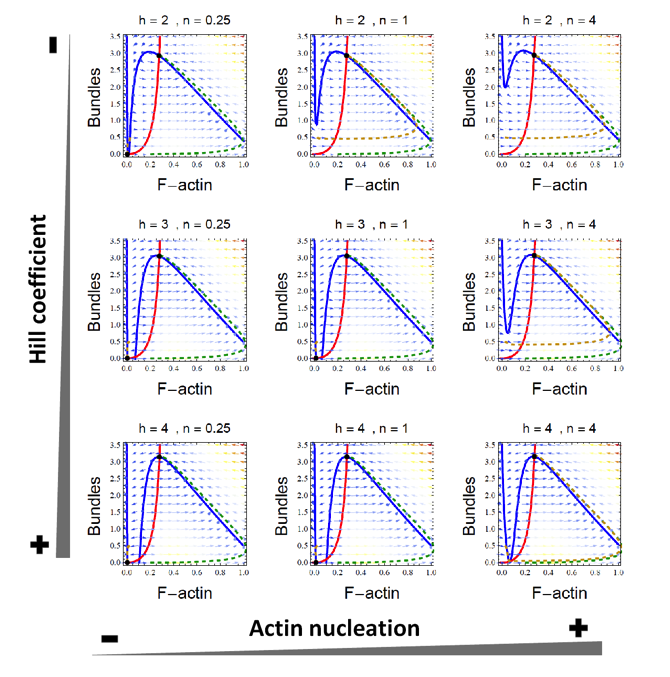

Supplement: S2 Fig — While the locations of the equilibrium points are altered, the qualitative relationship between the nullclines is unchanged. (TIF) [file pcbi.1005433.s004.tif]

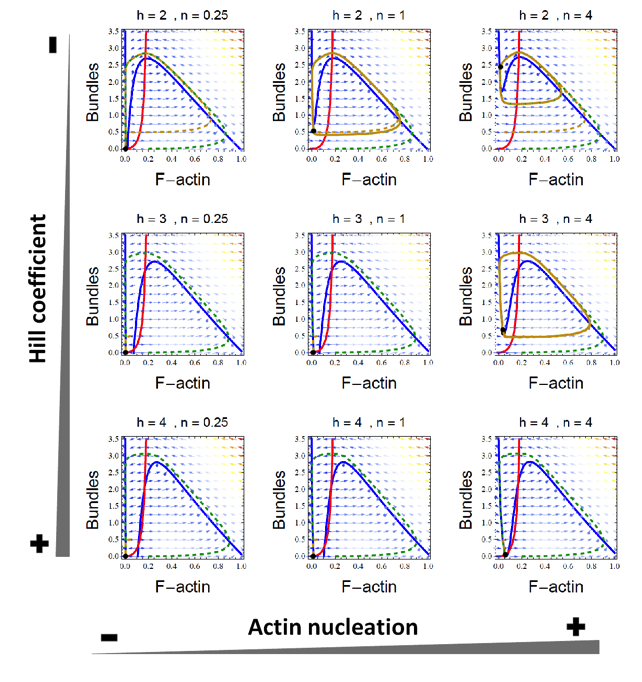

Supplement: S3 Fig — (TIF) [file pcbi.1005433.s005.tif]

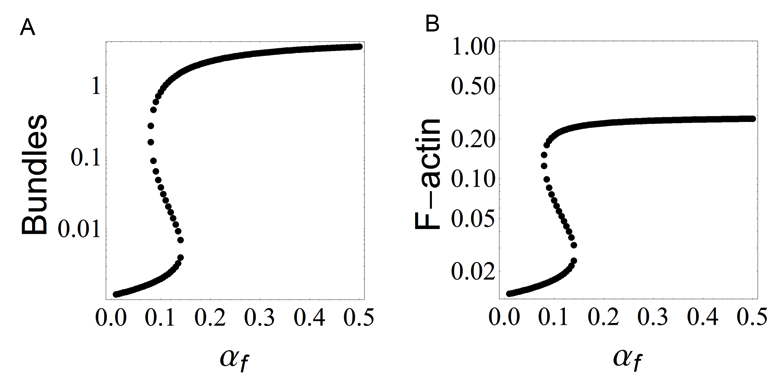

Supplement: S4 Fig — Other parameters as indicated in S1 Table. (TIF) [file pcbi.1005433.s006.tif]

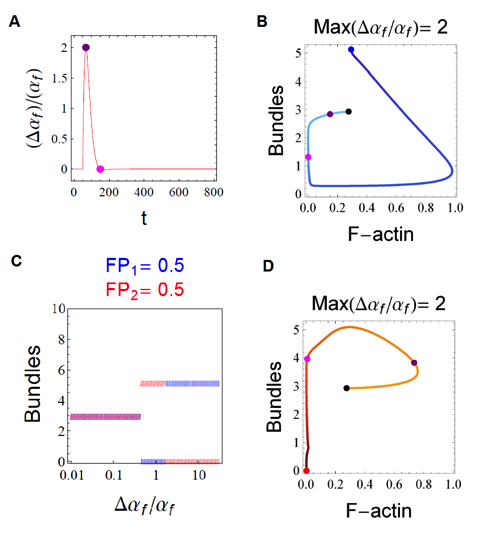

Supplement: S5 Fig — (A) Time course for transient stimulus imposed on the positive feedback αf for fraction FP2 or all FPs, and trajectories for concentrations of F-actin and bundles in the foot processes corresponding to regions FP1 (constant αf) and FP2 (transiently stimulated). (B) Trajectory for FP1. The time point of the peak and end of stimulus are represented in purple and magenta, respectively, in all plots. Time point zero is in black (at identical concentrations for FP1 and FP2) and steady-state value for each fraction is represented by shades of blue. (C) Steady state bundles in fractions FP1 (blue) and FP2 (red) as a function of stimulus intensity. (D) Trajectory for FP2. The time point of the peak and end of stimulus are represented in purple and magenta, respectively, in all plots. Time point zero is in black (at identical concentrations for FP1 and FP2) and steady-state value for each fraction is represented by shades of red. The intensity of the stimulus will alter the relative position between the two trajectories for unstimulated (FP1) and stimulated (FP2) fractions. Consequently, for sufficiently large perturbations, either region may collapse. (TIF) [file pcbi.1005433.s007.tif]

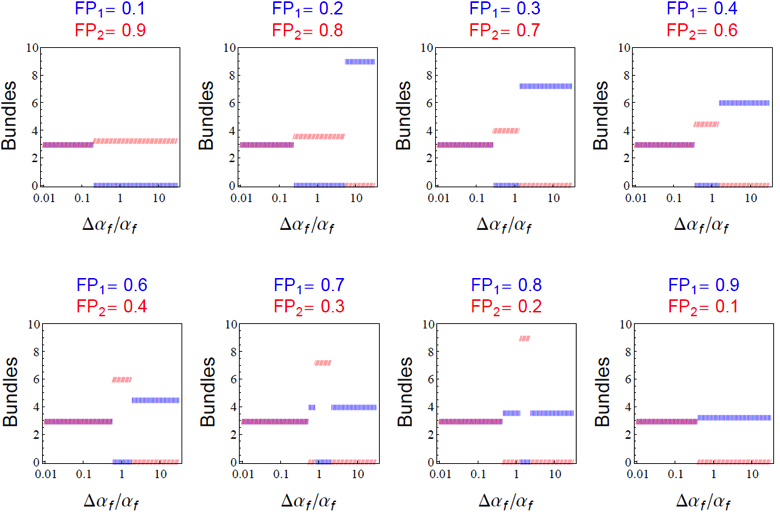

Supplement: S6 Fig — Over a broad range of fractions of FP1 and FP2 either region of the cell is subject to damage (collapse of bundles) if the perturbation is sufficiently strong. (TIF) [file pcbi.1005433.s008.tif]

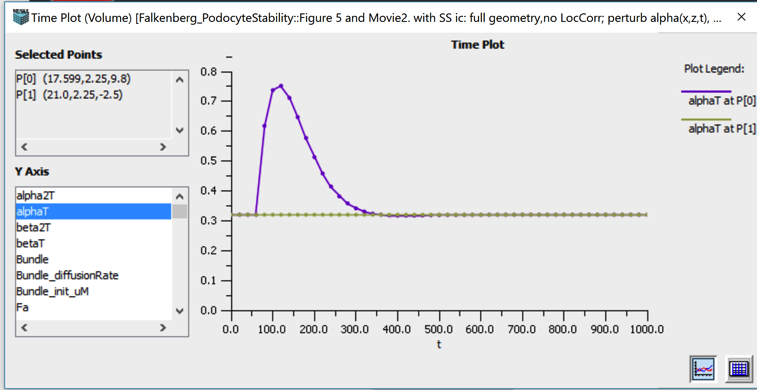

Supplement: S7 Fig — The spatial results for bundle concentration are shown in Fig 5. Nomenclature for parameters is described in S2 Table. (TIF) [file pcbi.1005433.s009.tif]

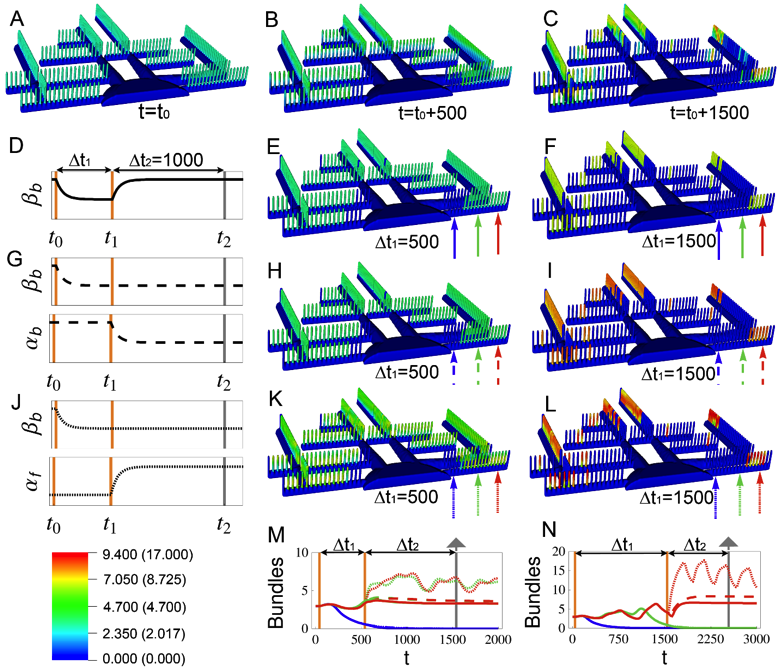

Supplement: S8 Fig — (A) Initial concentration of bundles at t = t0 where βb is reduced. The result is heterogeneous loss of bundles in some FPs at times (B) t = t0 + 500 and (C) t = t0 + 1500. Three lower rows of panels show the three different scenarios under which the bundling could be modified after a finite time, t1 following injury: (D) the parameter βb recovers its original value and the stabilized FPs can be observed after (E) t1 = 500 or (F) t1 = 1500. (G) Parameter βb can be decreased to compensate after t1 and stabilized FPs can be observed at (H) t1 = 500 or (I) t1 = 1500. (J) Alternatively, increase in αf can also halt loss of bundles in FPs whereby stabilized FPs can be observed at (K) t1 = 500 or (L) t1 = 1500. We can visualize the timecourses for bundle concentrations in randomly selected FPs (as identified by color-coded arrows) at (M) t1 = 500 or (N) t1 = 1500. Line style follows the same pattern as arrows, and corresponds to value of a single voxel in the middle of the corresponding FP. All 3-D snapshots follow the same color scale shown in bottom left (except for L, represented with skewed scale in parentheses). Under all of these scenarios, an earlier intervention leads to markedly improved homogeneous restoration of bundles. This can be clearly seen by the difference between the early intervention within the middle column (E, H, K) and late intervention within the right column (F, I, L). (TIF) [file pcbi.1005433.s010.tif]
