# Supplementary material for: Fragility of foot process morphology in kidney podocytes arises from chaotic spatial propagation of cytoskeletal instability
Source: PLoS Comput Biol. 2017 Mar 16;13(3):e1005433. doi: 10.1371/journal.pcbi.1005433 (PMC5373631; doi:10.1371/journal.pcbi.1005433)
Supplement: S1 Table — The parameter k = 0.22. Values differing from the ones used in Fig 2C are in bold. All units are arbitrary. (PDF) [file pcbi.1005433.s002.pdf]

Table S1. Parameters used in model for each figure, as in equations 1-3. The parameter k = 0.22. Values differing from the ones used in Fig 2c are in bold. All units are arbitrary.

**Figure**	α_f	α_b	β_f	β_b	γ_f	γ_b	Total actin
2C	0.32	0.03	0.15	0.01	0.001	0.0005	1
2D	**0.1**	0.03	0.15	0.01	0.001	0.0005	1
2E	0.32	0.03	0.15	**0.005**	0.001	0.0005	1
2F	**0.1**	0.03	0.15	**0.005**	0.001	0.0005	1
3	0.32	0.03<br>**0.05** (b, c)	0.15	0.01	0.001	0.0005	1<br>**0.7** (e)<br>**1.15** (f)
4	0.32	0.03	0.15	0.01	0.001	0.0005	1
5	0.32	0.03	0.15	0.01	0.001	0.0005	1
6<br>Min<br>Max	0.32<br><br>**2.00** (i, j)	0.03<br>**0.015** (f, g)	0.15	0.01<br>**0.005** (d, f, g, i, j)	0.001	0.0005	1
