# Supplementary material for: Fragility of foot process morphology in kidney podocytes arises from chaotic spatial propagation of cytoskeletal instability
Source: PLoS Comput Biol. 2017 Mar 16;13(3):e1005433. doi: 10.1371/journal.pcbi.1005433 (PMC5373631; doi:10.1371/journal.pcbi.1005433)
Supplement: S2 Table — (PDF) [file pcbi.1005433.s003.pdf]

Table S2. Nomenclature used in the spatial VCell model.

VCell	Manuscript symbol and function
Bundle	Bu, bundles
Fa	Fa, F-actin
Ga	Ga, G-actin
Loc	localization constant: positive feedback is limited to foot processes
Loc1	sub region, used to help define geometrical region of Loc
locCorrection	sub region, used to help define geometrical region of Loc in some simulations
perturb1	region where first geometric perturbation is applied
perturb2	region where second geometric perturbation is applied
skeleton	sub region, used to help define geometrical region of Loc
skeleton2	sub region, used to help define geometrical region of Loc
C0	constant, needed to ensure the model is stoichiometric
C1	constant, needed to ensure the model is stoichiometric
C2	constant, needed to ensure the model is stoichiometric
alphaT	parameter a_f, as a function of time
alpha2T	parameter a_b, as a function of time
betaT	parameter b_f, as a function of time
beta2T	parameter b_b, as a function of time
