# Supplementary material for: Fragility of foot process morphology in kidney podocytes arises from chaotic spatial propagation of cytoskeletal instability
Source: PLoS Comput Biol. 2017 Mar 16;13(3):e1005433. doi: 10.1371/journal.pcbi.1005433 (PMC5373631; doi:10.1371/journal.pcbi.1005433)

"RC 1"

nodes	→	distance, μm
(1,2)	→	8.25
(1,3)	→	17.65
(1,4)	→	7.09
(3,5)	→	8.15
(3,6)	→	6.87
(3,7)	→	6.11
(2,8)	→	41.03
(2,9)	→	10.06
(2,10)	→	12.74
(2,11)	→	13.94
(4,12)	→	8.82
(4,13)	→	9.9
(13,14)	→	1.3
(14,15)	→	10.59
(14,16)	→	9.4
(13,17)	→	10.25
(4,18)	→	10.82
(4,19)	→	6.53
(19,20)	→	11.48
(19,21)	→	6.87
(21,22)	→	15.56
(21,23)	→	8.67
(10,24)	→	6.83
(10,25)	→	4.53
(9,26)	→	16.96
(9,27)	→	16.14
(27,28)	→	10.84
(27,29)	→	7.21
(9,30)	→	10.795
(9,32)	→	10.795
(9,31)	→	16.7
(32,33)	→	5.18
(32,34)	→	9.35
(32,35)	→	5.61
(30,36)	→	4.71
(36,37)	→	5.11
(36,38)	→	11.74
(30,39)	→	1.96
(39,40)	→	17.27
(39,41)	→	21.29
(30,42)	→	9.98
(42,43)	→	7.79
(42,44)	→	10.32

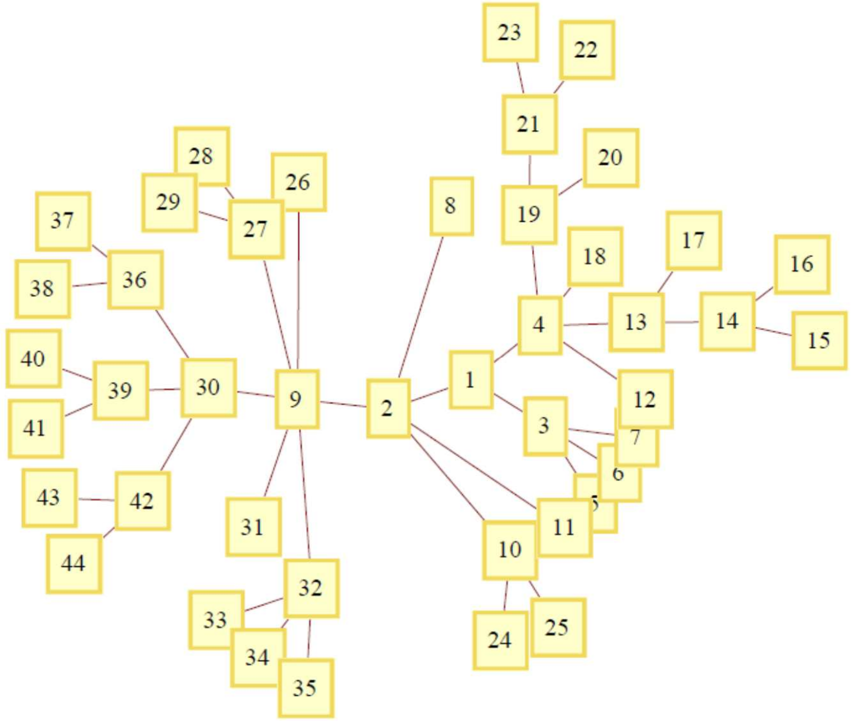

"RC 8"

nodes	→	distance, μm
(1,2)	→	11.53
(1,3)	→	16.3
(1,4)	→	8.82
(2,5)	→	7.31
(2,6)	→	8.61
(5,7)	→	6.69
(5,8)	→	7.99
(5,9)	→	12.31
(9,10)	→	8.56
(9,11)	→	8.81
(4,12)	→	19.23
(4,13)	→	17.38
(4,14)	→	16.36
(4,15)	→	9.11
(13,16)	→	9.93
(13,17)	→	6.6
(13,18)	→	6.05
(13,19)	→	5.64
(14,20)	→	10.59
(14,21)	→	21.51
(4,22)	→	6.22
(22,23)	→	1.89
(23,24)	→	18.34
(23,25)	→	13.65
(22,26)	→	6.99
(26,27)	→	10.28
(26,28)	→	6.65
(26,29)	→	6.25
(29,30)	→	9.15
(29,31)	→	13.19

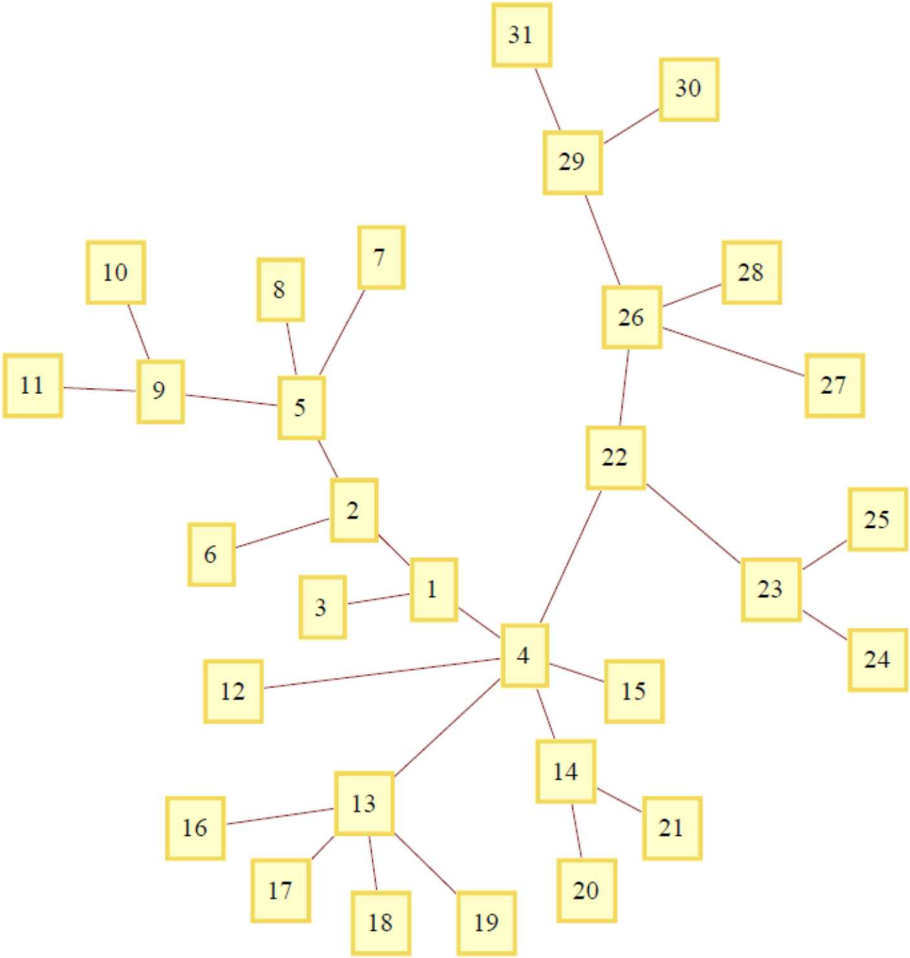

“RC 9”

nodes	→	distance, μm
(1,2)	→	13.32
(1,3)	→	13.02
(1,4)	→	10.34
(1,5)	→	5.01
(1,6)	→	6.3
(1,7)	→	21.09
(1,8)	→	21.72
(1,9)	→	24.15
(5,10)	→	21.77
(5,11)	→	24.01
(6,12)	→	24.48
(1,14)	→	18.41
(14,15)	→	1.87
(15,16)	→	30.08
(15,17)	→	12.58
(14,18)	→	9.67
(14,19)	→	16.8
(14,20)	→	15.58
(20,21)	→	5.31
(20,22)	→	9.54
(9,23)	→	13.17
(9,24)	→	6.86
(9,25)	→	6.46
(9,26)	→	8.61
(8,27)	→	9.61
(8,28)	→	7.38
(8,29)	→	2.97
(29,30)	→	7.03
(29,31)	→	4.11
(29,32)	→	6.66
(7,33)	→	9.56
(7,34)	→	8.35
(7,35)	→	7.99
(7,13)	→	5.28

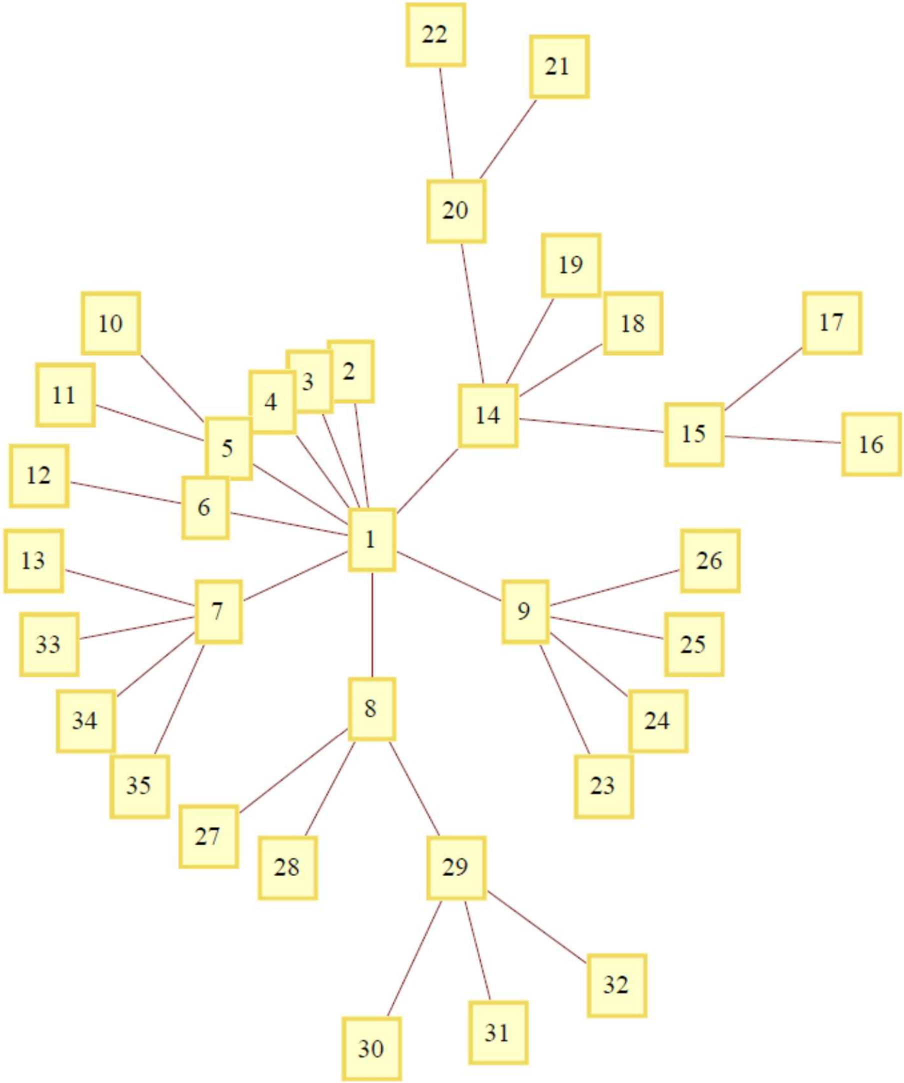

"RC 11"

nodes	→	distance, μm
(1,2)	→	1.87
(1,3)	→	2.09
(1,4)	→	16.15
(1,5)	→	22.82
(1,6)	→	12.69
(3,7)	→	18.51
(3,8)	→	14.56
(2,9)	→	13.02
(1,10)	→	16.92
(1,11)	→	17.62
(1,12)	→	11.18
(1,13)	→	14.88
(1,14)	→	5.94
(14,15)	→	14.94
(14,16)	→	15.34
(1,17)	→	15.31
(7,18)	→	6.87
(7,19)	→	6.73
(7,20)	→	4.25
(7,21)	→	4.91
(5,22)	→	9.38
(5,23)	→	7.25
(23,24)	→	16.83
(23,25)	→	12.99
(23,26)	→	12.55
(23,27)	→	10.33
(4,28)	→	11.57
(4,29)	→	8.53
(4,30)	→	6.53
(30,31)	→	3.27
(30,32)	→	12.18

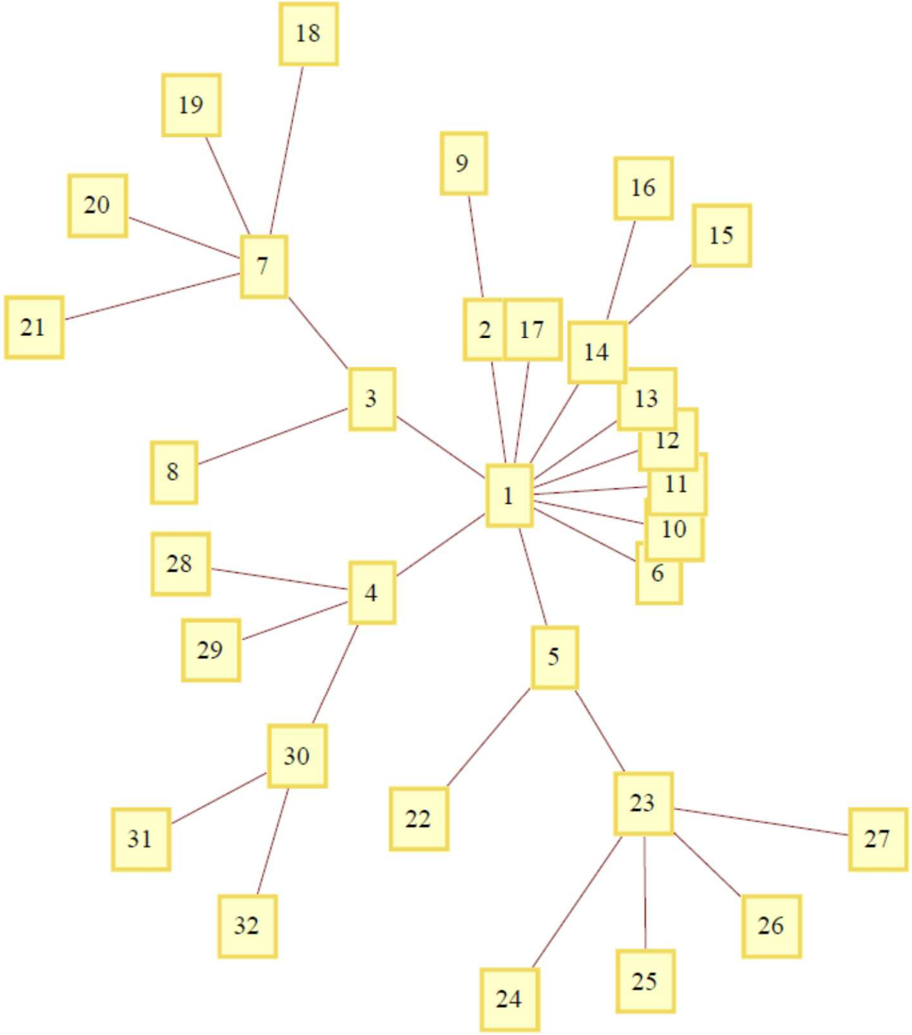

"RC 13"

nodes	→	distance, μm
(1,2)	→	21.73
(1,3)	→	23.92
(1,4)	→	15.09
(1,5)	→	19.06
(1,6)	→	38.28
(1,7)	→	17.92
(1,8)	→	21.55
(8,9)	→	5.7
(9,10)	→	7.59
(9,11)	→	5.19
(2,12)	→	5.63
(2,13)	→	5.25
(13,14)	→	5.11
(13,15)	→	4.
(4,16)	→	1.9
(16,17)	→	9.16
(16,18)	→	10.8
(5,19)	→	9.36
(19,20)	→	9.38
(19,21)	→	3.97
(5,22)	→	8.73
(22,23)	→	8.26
(22,24)	→	9.1
(5,25)	→	13.09
(25,26)	→	6.91
(25,27)	→	11.65
(5,28)	→	3.66
(28,29)	→	14.67
(28,30)	→	13.03
(30,31)	→	5.7
(30,32)	→	7.98
(32,33)	→	6.81
(32,34)	→	8.91
(4,35)	→	6.38

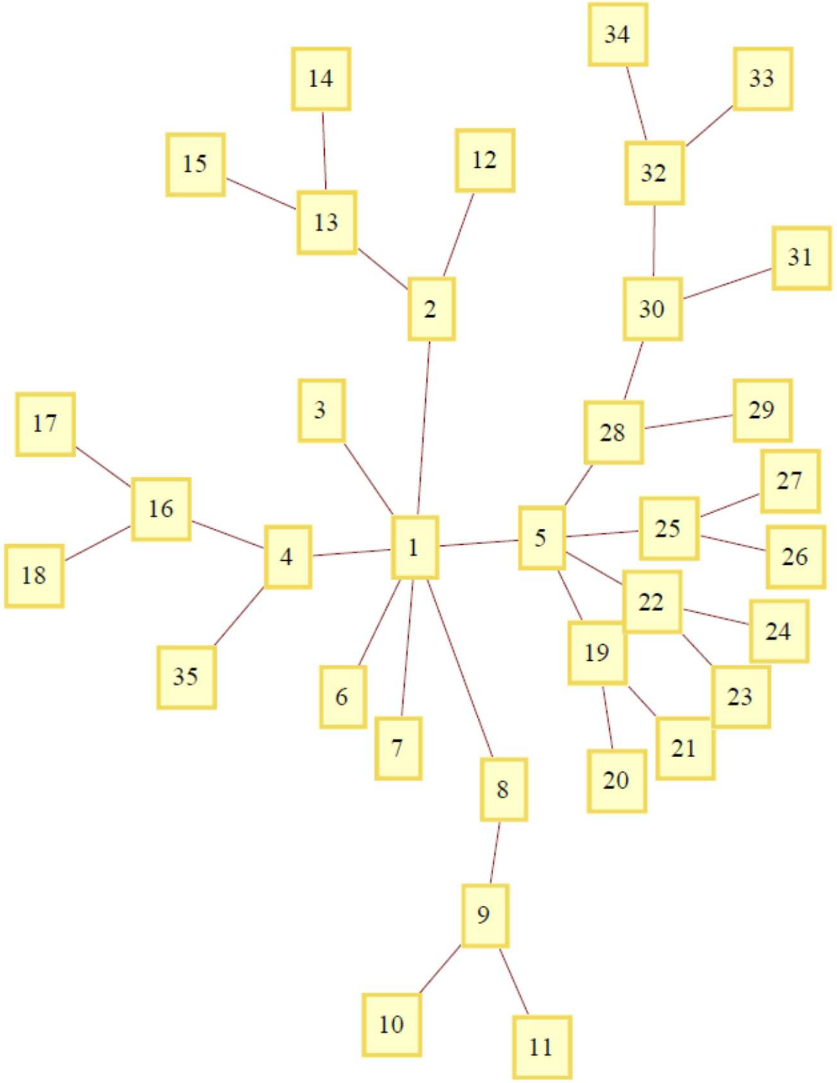

Supplement: S1 Dataset — (PDF) [file pcbi.1005433.s014.pdf]
