# Supplementary material for: Fragility of foot process morphology in kidney podocytes arises from chaotic spatial propagation of cytoskeletal instability
Source: PLoS Comput Biol. 2017 Mar 16;13(3):e1005433. doi: 10.1371/journal.pcbi.1005433 (PMC5373631; doi:10.1371/journal.pcbi.1005433)
Supplement: S1 Supplementary Materials and Methods — (PDF) [file pcbi.1005433.s015.pdf]

Supporting Materials and Methods

Fragility of foot process morphology in kidney podocytes arises from chaotic spatial propagation of cytoskeletal instability

Cibele V. Falkenberg*, Evren U. Azeloglu*, Mark Stothers, Thomas J. Deerinck, Yibang Chen, John C. He, Mark H. Ellisman, James C. Hone, Ravi Iyengar, and Leslie M. Loew

Methods for analysis of the podocyte morphology:

All animal procedures were approved by both Mount Sinai and UCSD Institutional Animal Care and Use Committees. Adult male 8-week-old Sprague-Dawley rats were anesthetized with ketamine-xylazine and perfused with 2.5% glutaraldehyde and 2% paraformaldehyde mixture in sodium cacodylate buffer. Kidneys were excised, cut into 2 x 2 x 2 mm^3 chunks, and processed successively with ferrocyanide-reduced osmium tetroxide followed by thiocardohydrazide-osmium liganding for increased contrast. After overnight incubation with uranyl acetate, tissues were stained *en bloc* with Walton's lead aspartate staining, dehydrated, and embedded in Durcupan ACM resin on Gatan aluminum sample pins. Edges were grounded with silver paint and the sample was sputter coated with palladium. Imaging was performed with low-voltage backscatter electrons using a Gatan 3View SBF-SEM system installed in a FEI Quanta 200 FEG scanning electron microscope using a 8000 x 8000 pixel raster with block-face images (Fig 1A).

The voxel size of the imaged cells was originally 0.011 x 0.011 x 0.210 μm^3. Due to memory limitations, none of the software used - Seg3D (Center for Integrative Biomedical Computing at University of Utah, Salt Lake City, Utah, USA), Imaris (Bitplane, Zurich, Switzerland), Virtual Cell (Center for Cell Analysis and Modeling at University of Connecticut, Farmington, CT, USA) - was able to handle the 3D images at full resolution. Each x-y slice was reduced to 10% using Adobe Photoshop CS3. The surface area and volume of the different regions of the cell were computed using Seg3D. Different filters allowed for the reconstruction of a full cell (yellow surface in Fig 1E) and a "FP-free" cell (blue surface in Fig 1E). The disparity in z-resolution was rectified by smoothing: the sequence of blur by four pixels followed by resample by 1-fold in z-direction was repeated twice. The volume and surface areas of the complete cell was computed after building a surface using the smoothed images. In order to identify the contribution of the FPs to volume and surface area of the cells, a second surface was built, without the FPs. It was obtained after applying a threshold filter of 50% (Fig 1E).

The core was estimated applying an analogy to a well-known heat transfer problem: the relationship between the body conductivity and the removal rate at the surface dictates whether the material will present uniform or graded temperature [22]. We performed a similar computation using VCell, making use of the mathematical equivalence between heat and mass diffusion processes. We used a uniform volumetric source term (0.04 μM/sec) in cells with diffusion coefficient 10μm^2/sec, and removal at cell walls (h = 0.04 $\mu m\ sec^{-1}$, flux proportional to the local value). As expected, regions of the cell with higher surface to volume ratios had higher 'losses' through the membrane, presented larger gradients and

lower values for concentrations. At steady-state, the volumetric region with concentration within 90% of the maximum was exported as an nrrd file. This volume was assigned as the core region (Fig 1F-1G). Its volume and surface areas were then computed using Seg3D. The remaining volume (after exclusion of FPs and core from the full cell) corresponds to primary and secondary processes. The core volume ranges to from 30 to 50% of the cell volume. With FPs corresponding to 20% of the volume, the remaining 50% to 30% of the volume corresponds to primary and secondary processes.

The distances between the center of the cell body and branch points or endpoints of the cell processes were measured using the filament tool in Imaris. The 'center' of the cell body was identified automatically, as the region of the cell with largest cross section. Branch points and end points were manually positioned, after careful examination of each cell (Fig 1I). For the five cells analyzed, the average distance from the center to a branch point was 20 ± 10 μm. Because we wanted to construct a quarter of a cell utilizing two axes of symmetry, we also collected the average distance between the center and the 12 furthest endpoints per cell: 38 ± 4 μm. Each cell presented a different branching pattern. The branching for two different cells are illustrated in Fig S1, tables with nodes and relative distances for five different cells can be found in Supporting Dataset S1. The lack of uniformity of the cell body lengths and branch distributions is also reflected in the analysis of volumetric properties of the core and major processes in Table 1, where standard deviations were larger than 20%. However, the geometric properties of the FPs seem to be tightly regulated, with a very narrow distribution of volume fraction relative to the total cell of 20% and with a very consistent surface to volume ratio of 9 μm^{-1}.

The analytical geometry was constructed with the objective of recapitulating the cell properties that are of relevance for reaction-diffusion equations (length, surface, and volume) rather than visual similarity. The application of symmetry allowed us to reduce the mesh needed for numerical discretization and computational cost. FPs emanate from major processes in a symmetric fashion, generally with equal number of FPs in each side of the process of origin. Therefore in our analytical geometry, the major processes are generated as halves, using the sagittal plane as a reflexive boundary condition. The FPs emanate perpendicularly to such boundary. Since there was no signature branching pattern for these cells, we also imposed axial symmetry. Consequently, the analytically constructed geometry produces simulations of a full podocyte cell, but at ¼ the size.

The constructed geometry was initially built taking into account only core and major processes. Once the surface to volume (see Table 1) and distances (18 ± 6 μm average distance to branch point, and 39 ± 2 μm for the three furthest endpoints) satisfied the analysis described above, the FPs were added. The quarter of the cell body (core plus major processes) has volume of 420 μm^3, surface area 695 μm^2. After 233 FPs were added, the volume corresponds to 530 μm^3 and surface area of 1683 μm^2 (Fig 1J). In comparison to Table1, the volumetric properties of the constructed geometry correspond to a good representation of the analyzed cells. The surface area of the constructed FPs is on the upper range of the analyzed values. This is likely a conservative estimate: the resolution of the acquired images is of the length scale of the FPs, and a loss of surface detail is expected. The computational model described next only uses volumetric variables, and is not affected by this assumption.

Numerical simulations:

All simulations of systems of ordinary differential equations were performed and plotted using Mathematica (Champaign, IL) using equations 1-3 or others as described in the next sections. The parameters used to generate all figures in the text are reported in Table S1. All spatial simulations were integrated using Virtual Cell (University of Connecticut, CT).

Table S1. Parameters used in model for each figure, as in eqs. 1-3. The parameter k=0.22. Values differing from the ones used in Fig 2c are in bold. All units are arbitrary.

Figure	α_f	α_b	β_f	β_b	γ_f	γ_b	Total actin
2c	0.32	0.03	0.15	0.01	0.001	0.0005	1
2d	**0.1**	0.03	0.15	0.01	0.001	0.0005	1
2e	0.32	0.03	0.15	**0.005**	0.001	0.0005	1
2f	**0.1**	0.03	0.15	**0.005**	0.001	0.0005	1
3	0.32	0.03<br>**0.05** (b, c)	0.15	0.01	0.001	0.0005	1<br>**0.7** (e)<br>**1.15** (f)
4	0.32	0.03	0.15	0.01	0.001	0.0005	1
5	0.32	0.03	0.15	0.01	0.001	0.0005	1
6<br>Min<br>Max	0.32<br><br>**2.00** (i, j)	0.03<br>**0.015** (f, g)	0.15	0.01<br>**0.005** (d, f, g, i, j)	0.001	0.0005	1

Spatial model in Virtual Cell:

Virtual Cell (VCell, University of Connecticut, CT) is freely available (http://vcell.org/) and the spatial simulations in this paper are available in the public model "Falkenberg_PodocyteStability". The relationship between the VCell model nomenclature and the manuscript are summarized in table S2. The figures and movies correspond to "Applications" within the VCell model, and they are explicitly named for clarity. Extra simulations that do not appear in the manuscript are also available with the label "extra". Applications and corresponding simulations for a much smaller geometry (only four foot processes attached to a 'cell body' are also available), identified by the label 'few fingers'. Those simulation results are consistent with the ones for the more complex geometry. 3-D images and movies were generated after exporting the VCell results as VTK unstructed files that were visualized as 3-D plots and saved using Visit (Lawrence Livermore National Lab, CA).

Table S2. Nomenclature used in the spatial VCell model.

Vcell	Manuscript symbol and function
Bundle	Bu, Bundles
Fa	Fa, F-actin
Ga	Ga, G-actin
Loc	localization constant: positive feedback is limited to foot processes
Loc1	sub region, used to help define geometrical region of Loc
locCorrection	sub region, used to help define geometrical region of Loc in some simulations
perturb1	region where first geometric perturbation is applied
perturb2	region where second geometric perturbation is applied
skeleton	sub region, used to help define geometrical region of Loc
skeleton2	sub region, used to help define geometrical region of Loc
C0	constant, needed to ensure the model is stoichiometric
C1	constant, needed to ensure the model is stoichiometric
C2	constant, needed to ensure the model is stoichiometric
alphaT	parameter α_f, as a function of time
alpha2T	parameter α_b, as a function of time
betaT	parameter β_f, as a function of time
beta2T	parameter β_b, as a function of time

Parameter Sensitivity Analysis

As described in the text, for simplicity, it was assumed that nucleation and elongation had similar rates. Here, we show that altering the value of the nucleation using the parameter 'n', or the degree of cooperativity for the positive feedback using the Hill coefficient 'h', in equation S1 (compare with equation 1) will not change the properties of the system. The qualitative relationship between the nullclines is unaltered, and the same range of results described in Fig 2 can be reproduced.

$$\frac{d(Fa)}{dt} = \alpha_f \; Ga \left(\frac{Fa^h}{Fa^h + k^h}\right) + \gamma_f \; Ga \; (Fa + 2 \, \mathrm{n} \, Ga) - \alpha_b Fa(2 \, Fa + Bu) - \beta_f Fa \qquad \text{(S1)}$$

From Fig S2 we can appreciate that weak actin nucleation would increase the chances of collapse of the actin cytoskeleton (weak nucleation moves the blue F-actin nullcline down). Increasing the Hill coefficient moves the bundles nullcline (red) towards lower F-actin values. Figure S3 shows that even with altered actin nucleating and Hill coefficients, hyperactive bundling gives rise to cyclic behavior or complete collapse of the cytoskeleton. Most importantly, what Figures S2 and S3 show, is that the qualitative function of all parameters discussed in the main text are maintained (Fig 2B) regardless of numerical details such as the exact relationship between nucleation and elongation, or the cooperative strength of the positive feedback. In practice, each of these parameters is very important. Given our current constraints regarding the number of unknowns, the model is sufficiently detailed and robust to capture a good range of results.

Multicompartmental ODE model with two FP regions:

The parameter α_f in the fraction FP_2 is subject to a time-dependent stimulus. All other parameters, and parameters in the fraction FP_1 remain constant.

$$\frac{d(Fa_1)}{dt} = \alpha_f\ Ga\left(\frac{{Fa_1}^2}{{Fa_1}^2+k^2}\right) + \gamma_f\ Ga\ (Fa_1 + 2\ Ga) - \alpha_b Fa_1(2\ Fa_1 + Bu_1) - \beta_f Fa_1 \quad \text{(S2)}$$

$$\frac{d(Bu_1)}{dt} = \alpha_b Fa_1(2\ Fa_1 + Bu_1) + \gamma_b\ Ga\ Bu_1 - \beta_b Bu_1 \quad \text{(S3)}$$

$$\frac{d(Fa_2)}{dt} = (\alpha_f + \Delta\alpha_f(t))\ \ Ga\left(\frac{{Fa_2}^2}{{Fa_2}^2+k^2}\right) + \gamma_f\ Ga\ (Fa_2 + 2\ Ga) - \alpha_b Fa_2(2\ Fa_2 + Bu_2) - \beta_f Fa_2 \quad \text{(S4)}$$

$$\frac{d(Bu_2)}{dt} = \alpha_b Fa_2(2\ Fa_2 + Bu_2) + \gamma_b\ Ga\ Bu_2 - \beta_b Bu_2 \quad \text{(S5)}$$

$$\int_{cell} Ga\ dV + \int_{FP1} Fa_1\ dV + \int_{FP1} Bu_1\ dV + \int_{FP2} Fa_2\ dV + \int_{FP2} Bu_2\ dV = (Total\ actin) \quad \text{(S6)}$$

We first look at the transient response when the fraction of FPs with constant parameters is the same as the perturbed fraction: 50%. If the stimulus is weak, both FP_1 and FP_2 regions recover the uniform bundle strength (blue and red lines, respectively, in Fig 4C). For moderate stimulus, FP_1 collapses permanently and FP_2 reaches a new equilibrium with stronger bundles (Fig 4D). For very strong stimulus, the initial response is as expected, but at longer times, the bundles in FP_2 collapse while the bundles in FP_1 are enhanced Fig 4E). The phase-plane diagrams in Fig S5B-S5D help explain the switch. Initially, FPs belonging to both FP_1 and FP_2 have the same composition regarding bundles and F-actin (black point, Fig S4B and S4D, respectively, approximately at position (0.3, 3) in the phase planes. With the strong stimulus, the FP_2 consumes G-actin in order to develop more and more fibers (the FP_2 trajectory moves to the right in the phase plane Fig S4D), while the production rate of fibers for FP_1 become weaker than its turnover (due to decreased availability of G-actin, FP_1 trajectory moves to the left in the phase plane Fig S4B). With high fiber content, the bundle formation is accelerated, converting fibers into bundles (the purple time point identifies the peak of the stimulus). By the time the stimulus $\Delta\alpha_f$ is over (magenta time point), all FPs have very little F-actin. However, FP_1 is "ahead" of FP_2 in the trajectory towards the equilibrium point (compare with light brown trajectory in Fig 2C), reestablishing stability at the cost of FP_2.

Next, we show the different steady states reached for different fractions of FP_1 and FP_2 over a wide range of transient stimulus intensity in Fig S6. The results suggest that the cells can recover from moderate stimulus, regardless of the fraction of FPs affected. However, as the stimuli get stronger, either the perturbed region (FP_2) or the unperturbed region (FP_1) may collapse. The balance depends on the strength ($\Delta\alpha_f$) and spatial coverage (ratio of FP_1 to FP_2) of the stimulus.

Spatial Simulations:

The software Virtual Cell automatically transforms a system of ODEs into PDEs by assigning geometry to a deterministic application. Effectively, a diffusion term is added to each ODE, with the generic functional form $\nabla \cdot (D\ \nabla c)$, with "$c$" representing the diffusing variable (G-actin, F-actin or bundle). The non-dimensional diffusion coefficient used for G-actin was $D_G = 10$, and for F-actin was $D_F = 0.005$. Since Bundles are very large structures, by definition anchored by a large family of actin binding proteins, their diffusion coefficient was set to $D_B = 0.005$ in the geometric region within the FPs, and $D_B = 10^{-20}$ elsewhere. In place of the conservation equation eq. 3, the PDE for G-actin can be written:

$$\frac{d(Ga)}{dt} = -\alpha_f\ Ga \left(\frac{Fa^2}{Fa^2+k^2}\right) - \gamma_f\ Ga\ (Fa + 2\ Ga) + \beta_f Fa - \gamma_b\ Ga\ Bu + \beta_b Bu + \nabla.(D_G\ \nabla\ Ga) \quad \text{(S7)}$$

And the PDE's for F-actin and bundles:

$$\frac{d(Fa)}{dt} = \alpha_f\ Ga \left(\frac{Fa^2}{Fa^2+k^2}\right) + \gamma_f\ Ga\ (Fa + 2\ Ga) - \alpha_b Fa(2\ Fa + Bu) - \beta_f Fa + \nabla.(D_F\ \nabla\ Fa) \quad \text{(S8)}$$

$$\frac{d(Bu)}{dt} = \alpha_b Fa(2\ Fa + Bu) + \gamma_b\ Ga\ Bu - \beta_b Bu + \nabla.(D_{\boldsymbol{B}}\ \nabla\ Bu) \quad \text{(S9)}$$

The spatial simulation for transient localized increase in positive feedback α_f is shown in the main text in Fig 5. The transient stimulus was implemented in VCell model as reported in Fig S7.
