# Supplementary material for: Fragility of foot process morphology in kidney podocytes arises from chaotic spatial propagation of cytoskeletal instability
Source: PLoS Comput Biol. 2017 Mar 16;13(3):e1005433. doi: 10.1371/journal.pcbi.1005433 (PMC5373631; doi:10.1371/journal.pcbi.1005433)
Supplement: S1 Text — (DOCX) [file pcbi.1005433.s016.docx]

**3-D Rat Podocyte Morphologies from SBF-SEM**

*Data from:*

**Fragility of foot process morphology in kidney podocytes arises from chaotic spatial propagation of cytoskeletal instability**

Cibele V. Falkenberg*, Evren U. Azeloglu*, Mark Stothers, Thomas J. Deerinck, Yibang

Chen, John C. He, Mark H. Ellisman, James C. Hone, Ravi Iyengar, and Leslie M. Loew

Kidney podocyte’s function depends on finger-like projections (foot processes) that interdigitate with those from neighboring cells to form the glomerular filtration barrier. The integrity of the barrier depends on spatial control of dynamics of actin cytoskeleton in the foot processes. We determined how imbalances in regulation of actin cytoskeletal dynamics can result in pathological morphology. We obtained 3-D electron microscopy images of podocytes and used quantitative features to build dynamical models to investigate how regulation of actin dynamics within foot processes controls local morphology. We find that imbalances in regulation of actin bundling, lead to chaotic spatial patterns that could impair the foot process morphology. Simulation results are consistent with experimental observations for cytoskeletal reconfiguration by dysregulated RhoA or Rac1, and they predict compensatory mechanisms for biochemical stability. We conclude that podocyte morphology, optimized for filtration, is intrinsically fragile, whereby local transient biochemical imbalances may lead to permanent morphological changes associated with pathophysiology.

This data package contains the segmented binary images (Level 1) and the Gaussian-smoothened reconstructed volumes (Level 2) of individual rat kidney podocytes that were used to create our dynamical models. Images have 11 nm/pixel in-plane (XY)-resolution and 210 nm out-of-plane (Z)-resolution. Reconstructed volumes all use a single coordinate system. Technical details of segmentation and reconstruction are provided in the Supplementary Information file associated with the manuscript.

**Structure of Raw Imaging Data**

**Level 0 (available at request)**

Raw images from Gatan SBF-SEM

Glomerulus of adult 8-week old healthy male Sprague Dawley rat; scanned June 16, 2011.

419 8-bit grayscale TIFF images @ 8,000 x 8,000 resolution (26.83 GB)

**Level 1 (available within this data package)**

Zip files for the segmented images from five control podocytes (116 MB)

RC-01: 101 binary JPG images (26.1 MB)

RC-08: 132 binary JPG images (17.6 MB)

RC-09: 133 binary JPG images (28.4 MB)

RC-11: 139 binary JPG images (18.7 MB)

RC-13: 111 binary JPG images (24.7 MB)

**Level 2 (available within this data package)**

STL files reconstructed using VCELL for five control podocytes (214 MB)

RC-01 (69.1 MB)

RC-08 (34.7 MB)

RC-09 (50.7 MB)

RC-11 (20.5 MB)

RC-13 (38.6 MB)
